# Medium-chain fatty acids suppress lipotoxicity-induced hepatic fibrosis via the immunomodulating receptor GPR84

**DOI:** 10.1172/jci.insight.165469

**Published:** 2023-01-24

**Authors:** Ryuji Ohue-Kitano, Hazuki Nonaka, Akari Nishida, Yuki Masujima, Daisuke Takahashi, Takako Ikeda, Akiharu Uwamizu, Miyako Tanaka, Motoyuki Kohjima, Miki Igarashi, Hironori Katoh, Tomohiro Tanaka, Asuka Inoue, Takayoshi Suganami, Koji Hase, Yoshihiro Ogawa, Junken Aoki, Ikuo Kimura

**Affiliations:** 1 Laboratory of Molecular Neurobiology, Graduate School of Biostudies and; 2 Laboratory of Molecular Neurobiology, Graduate School of Pharmaceutical Sciences, Kyoto University, Sakyo-ku, Kyoto, Japan.; 3 Department of Applied Biological Science, Graduate School of Agriculture, Tokyo University of Agriculture and Technology, Fuchu, Tokyo, Japan.; 4 Division of Biochemistry, Faculty of Pharmacy and Graduate School of Pharmaceutical Science, Keio University, Tokyo, Japan.; 5 Graduate School of Pharmaceutical Sciences, The University of Tokyo, Bunkyo-ku, Tokyo, Japan.; 6 Department of Molecular Medicine and Metabolism, Research Institute of Environmental Medicine, Nagoya University, Nagoya, Japan.; 7 Department of Medicine and Bioregulatory Science, Graduate School of Medical Sciences, Kyushu University, Fukuoka, Japan.; 8 Department of Gastroenterology and Metabolism, Graduate School of Medical Sciences and Medical School, Nagoya City University, Nagoya, Japan.; 9 Graduate School of Pharmaceutical Sciences, Tohoku University, Sendai, Miyagi, Japan.; 10 International Research and Development Center for Mucosal Vaccines, The Institute of Medical Science, The University of Tokyo (IMSUT), Bunkyo-ku, Tokyo, Japan.

**Keywords:** Hepatology, Inflammation, G protein&ndash;coupled receptors, Macrophages

## Abstract

Medium-chain triglycerides (MCTs), which consist of medium-chain fatty acids (MCFAs), are unique forms of dietary fat with various health benefits. G protein–coupled 84 (GPR84) acts as a receptor for MCFAs (especially C10:0 and C12:0); however, GPR84 is still considered an orphan receptor, and the nutritional signaling of endogenous and dietary MCFAs via GPR84 remains unclear. Here, we showed that endogenous MCFA-mediated GPR84 signaling protected hepatic functions from diet-induced lipotoxicity. Under high-fat diet (HFD) conditions, GPR84-deficient mice exhibited nonalcoholic steatohepatitis (NASH) and the progression of hepatic fibrosis but not steatosis. With markedly increased hepatic MCFA levels under HFD, GPR84 suppressed lipotoxicity-induced macrophage overactivation. Thus, GPR84 is an immunomodulating receptor that suppresses excessive dietary fat intake–induced toxicity by sensing increases in MCFAs. Additionally, administering MCTs, MCFAs (C10:0 or C12:0, but not C8:0), or GPR84 agonists effectively improved NASH in mouse models. Therefore, exogenous GPR84 stimulation is a potential strategy for treating NASH.

## Graphical abstract

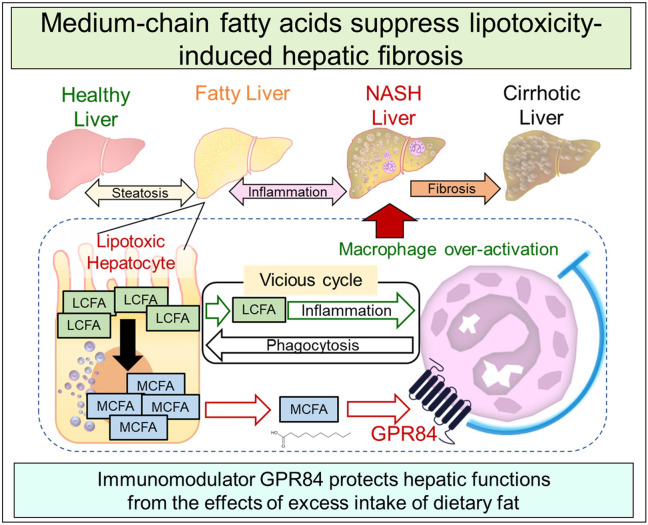

## Introduction

Nonalcoholic fatty liver disease (NAFLD) is the most common chronic liver disease worldwide (1–6). NAFLD includes a spectrum of well-defined stages, encompassing simple fatty liver (NAFL), which is a mostly benign condition, and nonalcoholic steatohepatitis (NASH). NASH progresses to cirrhosis and hepatocellular carcinoma (HCC) by activating inflammatory cascades and fibrogenesis (2, 3). The major risk factors of NASH include metabolic disorders such as obesity, insulin resistance, glucose intolerance or type 2 diabetes, and dyslipidemia (4, 5). Although the prevalence of NASH is rising in parallel with the global obesity pandemic, effective therapeutic strategies against the former are still in development (1, 6). Patients have to undergo liver transplantation to prevent the progression of NASH. The crucial event involved in NAFLD progression is hepatic lipotoxicity resulting from an excessive free fatty acid (FFA) influx from the peripheral tissues, mainly the adipose tissue, to hepatocytes or from increased hepatic de novo lipogenesis (1–5). Hepatic lipotoxicity occurs when the capacity of hepatocytes to manage and export FFAs as triglycerides (TGs) is overwhelmed.

FFAs act as energy sources and affect physiological functions such as hormone secretion, immune responses, and neurotransmission via the FFA-specific receptors FFAR1, FFAR4 (for long-chain fatty acids), FFAR2, and FFAR3 (for short-chain fatty acids) (7–12). Medium-chain fatty acids (MCFAs) also have a specific receptor — G protein–coupled receptor 84 (GPR84) (7, 13, 14). However, GPR84 is still considered an orphan G protein–coupled receptor (GPCR) because of the low plasma levels of endogenous MCFAs (15). Medium-chain triglycerides (MCTs), which consist of MCFAs, are unique forms of dietary fat exhibiting various health benefits (7, 16, 17). MCTs are an appropriate dietary choice for individuals with high energy demands. In the elderly, MCTs counteract age-related decreased energy production, and in athletes, MCTs enhance performance. MCTs are also beneficial for individuals who have undergone major surgeries or experience stunted growth (18–20). GPR84 is coupled with the pertussis toxin–sensitive Gi/o protein and is predominantly expressed in the BM, lungs, and peripheral leukocytes (13, 14, 21). Although some studies on GPR84-deficient mice have demonstrated that GPR84 plays an important role in immune and metabolic responses and may mediate the crosstalk between immune cells and adipocytes (22–25), comprehensive and integrated data bridging the gap between endogenous MCFAs and GPR84 are lacking, and the molecular mechanisms underlying these processes remain unclear.

Here, we investigated the effects of molecular nutritional signaling by MCFAs on metabolic functions using GPR84-deficient mice, a model of high-fat diet–induced (HFD-induced) obesity, and a NASH mouse model.

## Results

### GPR84 deficiency accelerates chronic inflammation under HFD conditions.

**Figure 1:**
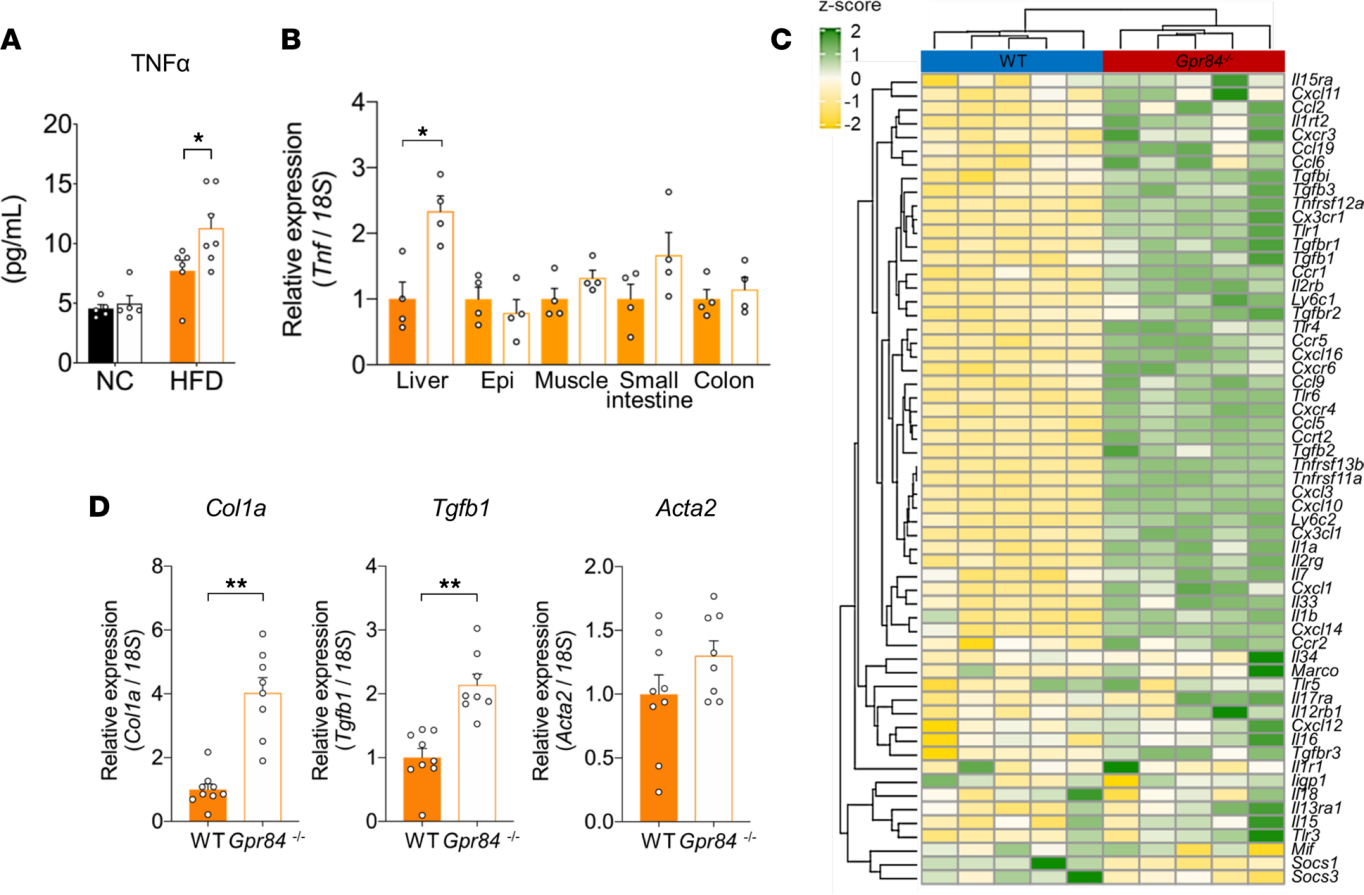
GPR84 deficiency accelerates HFD-induced chronic inflammation. (**A**) TNF-α levels (NC-fed group, *n* = 5; HFD-fed group, *n* = 6–7). NC, normal chow. (**B**) Expression of *Tnf* in liver, Epi, muscle, small intestine, and colon (*n* = 4 independent experiments). Data are represented as relative to the gene expression in WT mice. Epi, epididymal white adipose tissue. (**C**) RNA-Seq transcriptome profiling in liver in WT and *Gpr84^–/–^* mice fed the HFD for 5 weeks. Heatmap shows results of 2-dimensional hierarchical clustering of 59 genes related to inflammation (*n* = 5 per group). (**D**) Expression of fibrosis-related genes — *Col1a* (left), *Tgfb1* (middle), and *Acta2* (right) — in the liver (*n* = 8–9). Data are represented as relative to the gene expression in WT mice. *P < 0.05; **P < 0.01 (Mann-Whitney *U* test: **A**, **B**, and **D**). All data are presented as the mean ± SEM.

To study the role of GPR84 in the metabolic and immune systems, we generated *Gpr84^–/–^* mice (Supplemental Figure 1, A–C; supplemental material available online with this article; https://doi.org/10.1172/jci.insight.165469DS1). HFD feeding in WT mice increases the levels of inflammatory cytokines, such as TNF-α, and long-term HFD exposure leads to chronic inflammation (26). Therefore, we first compared the levels of plasma TNF-α as an inflammatory marker under short-term HFD feeding between WT and *Gpr84^–/–^* mice. Although plasma TNF-α levels were comparable between WT and *Gpr84^–/–^* mice under normal chow (NC) feeding, HFD feeding markedly increased plasma TNF-α levels in *Gpr84^–/–^* mice more than in WT mice (Figure 1A; 46.24% increase). Moreover, the hepatic expression of the *Tnf* mRNA in *Gpr84^–/–^* mice was markedly higher than that in WT mice, whereas its expression in other tissues, such as the white adipose tissue (WAT) (in both mature adipocytes and stromal vascular fraction), muscle, small intestine, and colon, was comparable between WT and *Gpr84^–/–^* mice (Figure 1B and Supplemental Figure 2). RNA-Seq and a Gene Ontology (GO) enrichment analysis of liver from the HFD-fed *Gpr84^–/–^* mice revealed a relationship between the chemokine pathway and chronic inflammation (Supplemental Figure 3, A–C). Among the differentially expressed genes (DEGs), the expression of 59 inflammation-related genes was altered compared with that of the WT mice (Figure 1C). In particular, the hepatic mRNA expression of the fibrosis markers *Col1a*, *Tgfb1*, and *Acta2* was considerably higher in *Gpr84^–/–^* mice than in WT mice (Figure 1D; *Col1a*: 4.03-fold increase, *Tgfb1*: 2.14-fold increase, and *Acta2*: 1.30-fold increase). The hepatic TG levels and mRNA expression of these fibrosis marker genes in the WAT were comparable between the groups (Supplemental Figure 4, A and B). Thus, GPR84-deficient mice exhibited chronic hepatic inflammation and fibrosis without the acceleration of hepatic fat accumulation, even under short-term HFD feeding.

### Long-term HFD-fed GPR84-deficient mice exhibit NASH.

**Figure 2:**
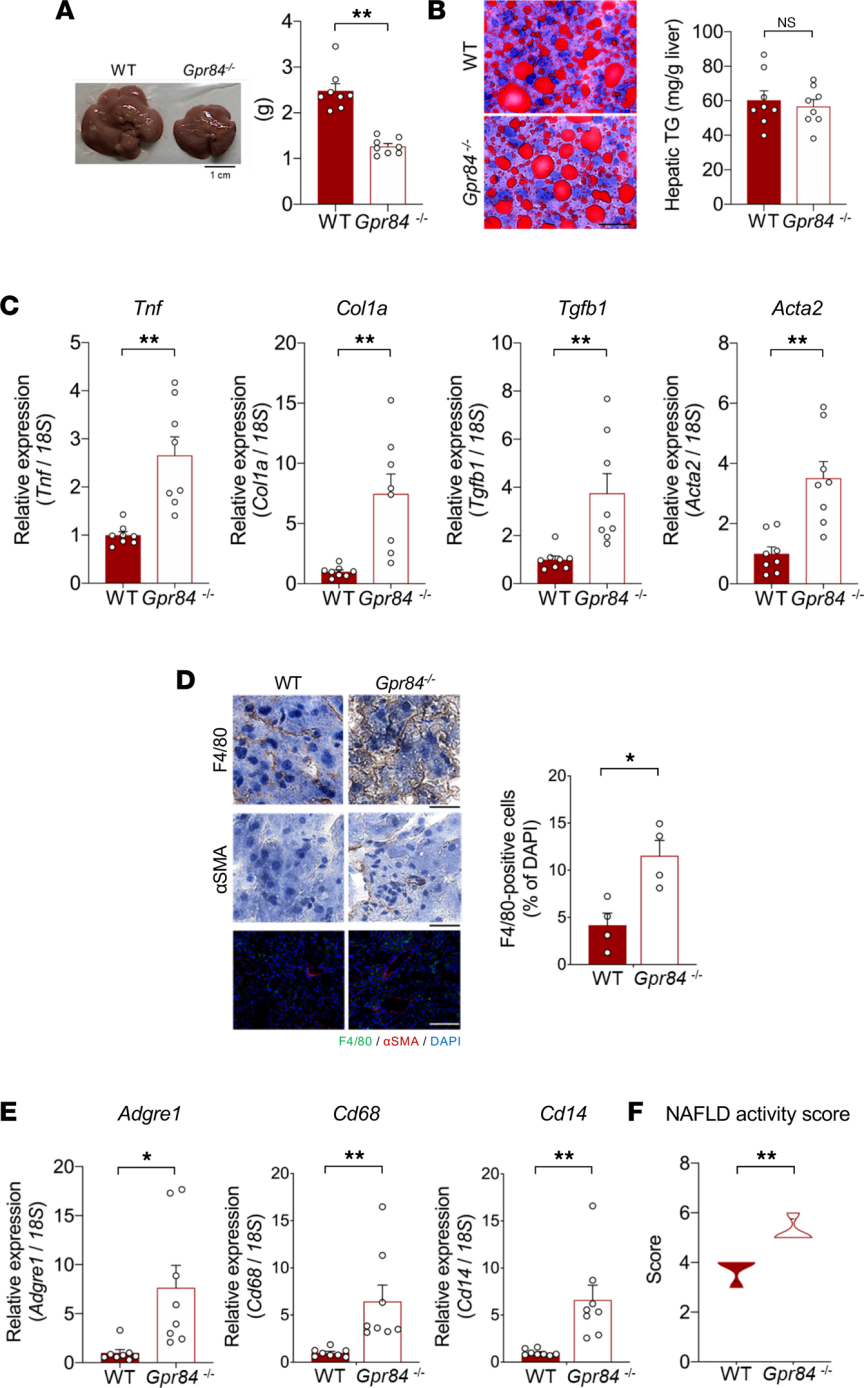
HFD-fed *Gpr84^–/–^* mice exhibit NASH. (**A**) Liver weight (*n* = 8 tissues per group). (**B**) Oil Red O staining (left) and hepatic TG levels (right) (*n* = 8 tissues per group). Scale bars: 25 μm. (**C**) Expression of *Tnf* and fibrosis marker genes — *Col1a* (left), *Tgfb1* (middle), and *Acta2* (right) — in WAT (*n* = 8 independent experiments). Data are represented as relative to the gene expression in WT mice. (**D**) IHC of F4/80 and α-SMA stained with DAB or fluorescence staining in sections of liver (left; F4/80, green; α-SMA, red; DAPI, blue). F4/80-positive cell numbers (right; *n* = 4 tissues per group). Scale bars: 25 μm (DAB staining) or 100 μm (fluorescence staining). (**E**) Expression of *Adgre1*, *Cd68*, and *Cd14* (*n* = 8 tissues per group). Data are represented as relative to the gene expression in WT mice. (**F**) NAS. *P < 0.05; **P < 0.01 (Mann-Whitney *U* test: **A**–**D**; Student’s *t* test: **E** and **F**). All data are presented as the mean ± SEM.

To determine how GPR84 deficiency affects the liver, we induced chronic inflammation and hepatic steatosis through long-term (12 weeks) feeding of an HFD to WT and *Gpr84^–/–^* mice. Liver weight was markedly lower in *Gpr84^–/–^* mice than in WT mice (Figure 2A), and the hepatic TG levels were comparable between them (Figure 2B). The hepatic levels of the inflammatory marker *Tnf* and the fibrosis markers *Col1a*, *Tgfb1*, and *Acta2* were markedly elevated in *Gpr84^–/–^* mice compared with those in WT mice (Figure 2C; *Tnf:* 2.66-fold increase, *Col1a*: 8.46-fold increase, *Tgfb1*: 3.76-fold increase, and *Acta2*: 3.51-fold increase), whereas their levels in WAT were comparable (Supplemental Figure 4, C and D). Furthermore, HFD-fed *Gpr84^–/–^* mice showed increased numbers of F4/80-positive macrophages, levels of the fibrosis marker α–smooth muscle actin (α-SMA) (Figure 2D), and the macrophage marker genes *Adgre1*, *Cd68*, and *Cd14* in the liver compared with HFD-fed WT mice (Figure 2E; *Adgre1*: 7.65-fold increase, *Cd68*: 6.87-fold increase, and *Cd14*: 6.75-fold increase). Consequently, the NAFLD activity score (NAS) for the livers of HFD-fed *Gpr84^–/–^* mice was higher than that for the livers of HFD-fed WT mice (Figure 2F). Thus, GPR84 deficiency accelerates the progression from HFD-induced hepatic steatosis to NASH.

### HFD feeding increases endogenous MCFA levels as GPR84 ligands in liver.

**Figure 3:**
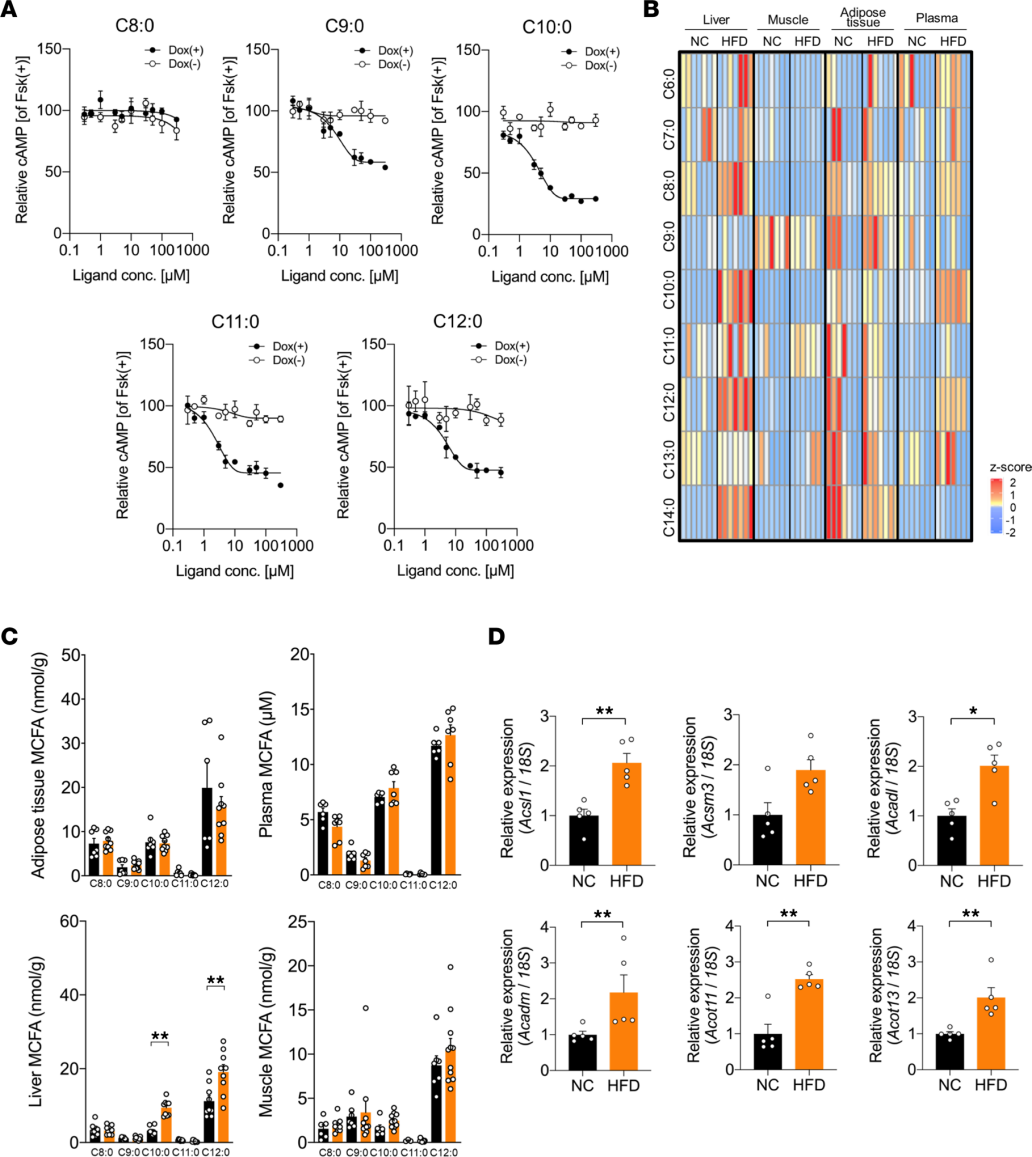
Affinity of MCFAs for GPR84 and RNA-Seq transcriptome profiling of liver under NC and HFD feeding. (**A**) cAMP inhibition assay for C8:0, C9:0, C10:0, C11:0, and C12:0 using mouse-GPR84–expressing HEK293 cells. Cells were cultured for 24 hours then treated with or without 10 μg/mL of Dox (*n* = 6 independent cultures with Dox, from 2 biological replicates; *n* = 6 independent cultures without Dox, from 2 biological replicates). All data are presented as relative to forskolin-induced (Fsk-induced) cAMP levels. Filled symbols represent values from cells treated with Dox, and unfilled symbols denote untreated groups. (**B**) Heatmap of relative MCFA contents among liver, muscle, adipose tissue, and plasma of WT mice after 5-week HFD feeding. (**C**) Measurement of MCFA concentration (NC-fed group, *n* = 6–9 tissues; HFD-fed group, *n* = 7–9 tissues). (**D**) Fatty acid synthesis– and β-oxidation–related genes were determined by real-time quantitative PCR (*n* = 5 from 5 per group). Data are represented as relative to the gene expression in NC-fed mice. *P < 0.05; **P < 0.01 (Mann-Whitney *U* test). All data are presented as the mean ± SEM.

GPR84 has been identified as a receptor for MCFAs and is coupled with the Gi/o protein, which decreases the intracellular cAMP concentration (14). In HEK293 cells expressing mouse GPR84 (Supplemental Figure 5A), C9:0, C10:0, C11:0, C12:0, and C13:0 activated GPR84 in a dose-dependent manner, whereas such activation was not displayed by C6:0, C7:0, C8:0, and C14:0 or not observed in doxycycline-uninduced controls (Dox-uninduced controls; non-GPR84–expressing HEK293 cells) (Figure 3A and Supplemental Figure 5B). C10:0 was found to be the most potent agonist of GPR84, with an EC_50_ of 3.5 μM, and C12:0 was the second-most potent agonist, with an EC_50_ of 4.4 μM (Figure 3A).

Next, we investigated the levels of endogenous MCFAs as GPR84 ligands after HFD feeding. As for the profiles of FFAs (C6:0–C14:0) including MCFAs, their levels were found to be elevated in the plasma and liver of HFD-fed mice compared with those in NC-fed mice (Figure 3B and Supplemental Figure 6A). The hepatic levels of C10:0 and C12:0 were markedly elevated in HFD-fed mice compared with those in NC-fed mice (Figure 3C). This increase in MCFA levels sufficiently activated GPR84 (Figure 3A). MCFAs were hardly detected in cecal contents under HFD conditions (Supplemental Figure 6, B and C). Comparison of the RNA-Seq data of the liver from NC- and HFD-fed mice showed that the 6 fatty acid synthesis and β-oxidation genes were coded as MCFA synthesis–related enzymes in 34 fatty acid synthesis and metabolism-related genes of DEGs (Supplemental Figure 6D). That is, acyl-CoA synthetase long-chain family member 1 (*Acsl1*) and acyl-CoA synthetase medium-chain family member 3 (*Acsm3*) code medium-chain acyl-CoA synthetase. Acyl-CoA dehydrogenase, long chain (*Acadl*), and acyl-CoA dehydrogenase, medium chain (*Acadm*), code medium-chain acyl-CoA dehydrogenase. Acyl-CoA thioesterase 11 (*Acot11*) and acyl-CoA thioesterase 13 (*Acot13*) code medium-chain acyl-CoA thioesterase. The hepatic mRNA expression levels of these MCFA synthesis-related enzymes were considerably higher in HFD-fed mice than in NC-fed mice (Figure 3D). Thus, HFD feeding increases the levels of endogenous MCFAs, which are GPR84 ligands, and accelerates fatty acid synthesis and β-oxidation in the liver.

### GPR84 suppresses BM-derived hepatic macrophages.

**Figure 4:**
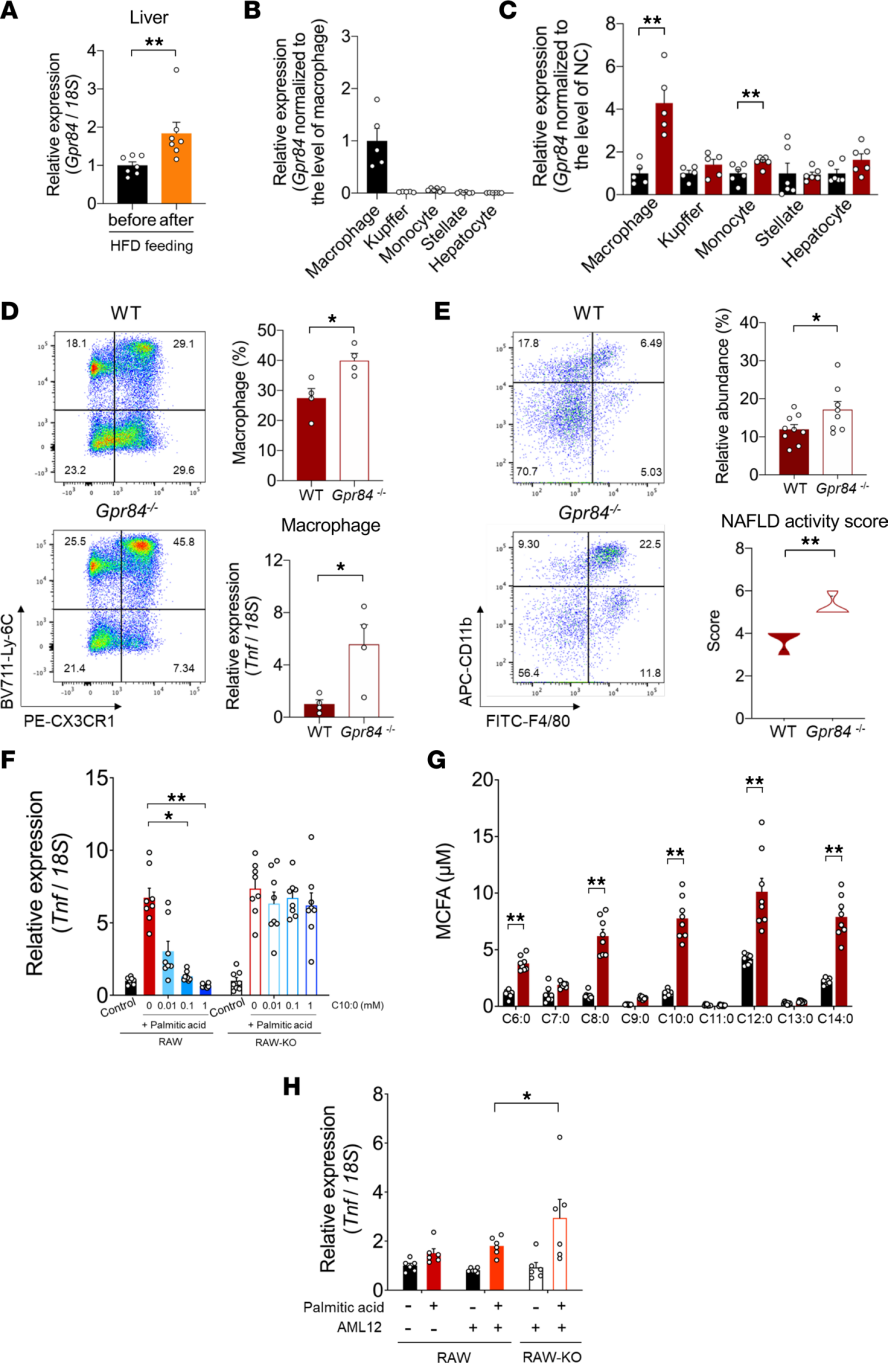
GPR84 suppresses BM-derived hepatic macrophages. (**A**) Expression of *Gpr84* in the liver after HFD feeding for 5 weeks (*n* = 7 tissues per group). Data are represented as relative to the gene expression in before HFD-fed mice. (**B**) *Gpr84* expression in BM-derived monocytes, hepatic macrophages, Kupffer cells, hepatic stellate cells, and hepatocytes isolated from WT mice fed NC for 12 weeks (*n* = 5–6 per group). Data are represented as relative to the gene expression in macrophage. (**C**) Change of *Gpr84* expression after HFD feeding (NC- vs. HFD-fed group, *n* = 5–6 per group). Data are represented as relative to the gene expression in NC-fed mice. (**D**) Flow cytometric analysis of BM-derived hepatic macrophage population and *Tnf* expression in WT and *Gpr84^–/–^* mice fed the HFD for 12 weeks (*n* = 4 per group). BM-derived hepatic macrophages, CD45^+^Ly6C^+^F4/80^+^CD11b^hi^CX3CR1^+^. (**E**) Flow cytometric analysis showing hepatic cell profile in BM-chimeric mice fed the HFD for 8 weeks (*n* = 8–9 per group) and NAS (*n* = 4 per group). WT recipient mice (CD45.1) after BM transplantation from WT or *Gpr84^–/–^* donor mice (CD45.2). (**F**) Antiinflammatory effect of MCFA-stimulated GPR84 (*n* = 8 per group; independent experiments). Data are represented as relative to the gene expression in untreated cells. (**G**) Intracellular MCFA production in AML12 cells (mouse hepatocyte cell line) treated with palmitic acid for 48 hours (*n* = 8 per group; independent experiments). (**H**) *Tnf* expression in RAW264.7 cells cocultured with AML12 prestimulated by palmitic acid (C16:0; *n* = 6 per group; independent experiments). *P < 0.05; **P < 0.01 (Mann-Whitney *U* test: **A** and **C**–**E** upper, **G**; Student’s *t* test: **E** lower; and Kruskal-Wallis with Dunn’s post hoc test: **F** and **H**). All data are presented as the mean ± SEM.

We next investigated the molecular mechanisms underlying the protective activity of GPR84 against the progression of HFD-induced hepatic steatosis to fibrosis. The HFD increased not only hepatic endogenous MCFA production but also hepatic *Gpr84* mRNA expression (Figure 4A). Hepatic *Gpr84* was expressed in macrophages but not in hepatocytes, monocytes, stellate, or Kupffer cells (Figure 4B), and HFD feeding further accelerated its expression (Figure 4C). The population of macrophages in the livers of HFD-fed *Gpr84^–/–^* mice was higher than that in HFD-fed WT mice (Figure 4D). In contrast, the population of macrophages in the livers of NC-fed *Gpr84^–/–^* mice was comparable to that of macrophages in the livers of WT mice (Supplemental Figure 7A). Additionally, the population of Kupffer cells in the livers of both NC- and HFD-fed *Gpr84^–/–^* mice were also comparable to that of Kupffer cells in the livers of WT mice (Supplemental Figure 7, A and B). Moreover, *Tnf* mRNA expression was markedly higher in the hepatic macrophages of HFD-fed (versus NC-fed) *Gpr84^–/–^* mice than in HFD-fed WT mice (Figure 4D and Supplemental Figure 7C). *Gpr84* was mainly expressed in the BM, which is the primary site of hematopoiesis (Supplemental Figure 7D). Hence, we further investigated the GPR84-mediated relationship between BM and hepatic macrophages. RNA-Seq and Gene Ontology (GO) enrichment analysis of the BM from HFD-fed *Gpr84^–/–^* mice showed that its expression profile was related to the macrophage-related chemokine pathway and chronic inflammation (Supplemental Figure 8, A–C). Additionally, the transplantation of *Gpr84^–/–^* mouse-derived BM into WT mice caused macrophage infiltration into the liver and NASH under HFD feeding as well as the hepatic phenotype of *Gpr84^–/–^* mice (Figure 4E). Thus, GPR84-positive BM-derived macrophages may prevent hepatic fibrosis.

The mechanisms underlying this process in the liver were investigated under HFD feeding conditions using GPR84-deficient RAW264.7 macrophages. Saturated fatty acids, such as palmitic acid (C16:0), which are abundant in HFD, induce inflammation by activating macrophages (7, 27). C16:0 stimulation upregulated the expression of the inflammatory marker *Tnf* and the macrophage infiltration marker CC chemokine ligand 2 (*Ccl2*) in RAW264.7 cells (Figure 4F and Supplemental Figure 9). C10:0 suppressed these effects and increased the expression of the antiinflammatory M2 macrophage marker arginase 1 (*Arg-1*) in a dose-dependent manner; while the effects of C10:0 were diminished in *Gpr84^–/–^* RAW264.7 cells (Figure 4F and Supplemental Figure 9). Furthermore, C16:0 administration increased the levels of intracellular MCFAs in the mouse hepatocyte cell line AML12 (Figure 4G). C16:0 stimulation in *Gpr84^–/–^* RAW264.7 cells cocultured with AML12 showed a marked increase in *Tnf* expression compared with that in RAW264.7 cells cocultured with AML12 (Figure 4H). Thus, MCFAs suppress lipotoxicity-induced macrophage activation via GPR84 in the liver.

### GPR84 activation by MCFAs improves NASH.

**Figure 5:**
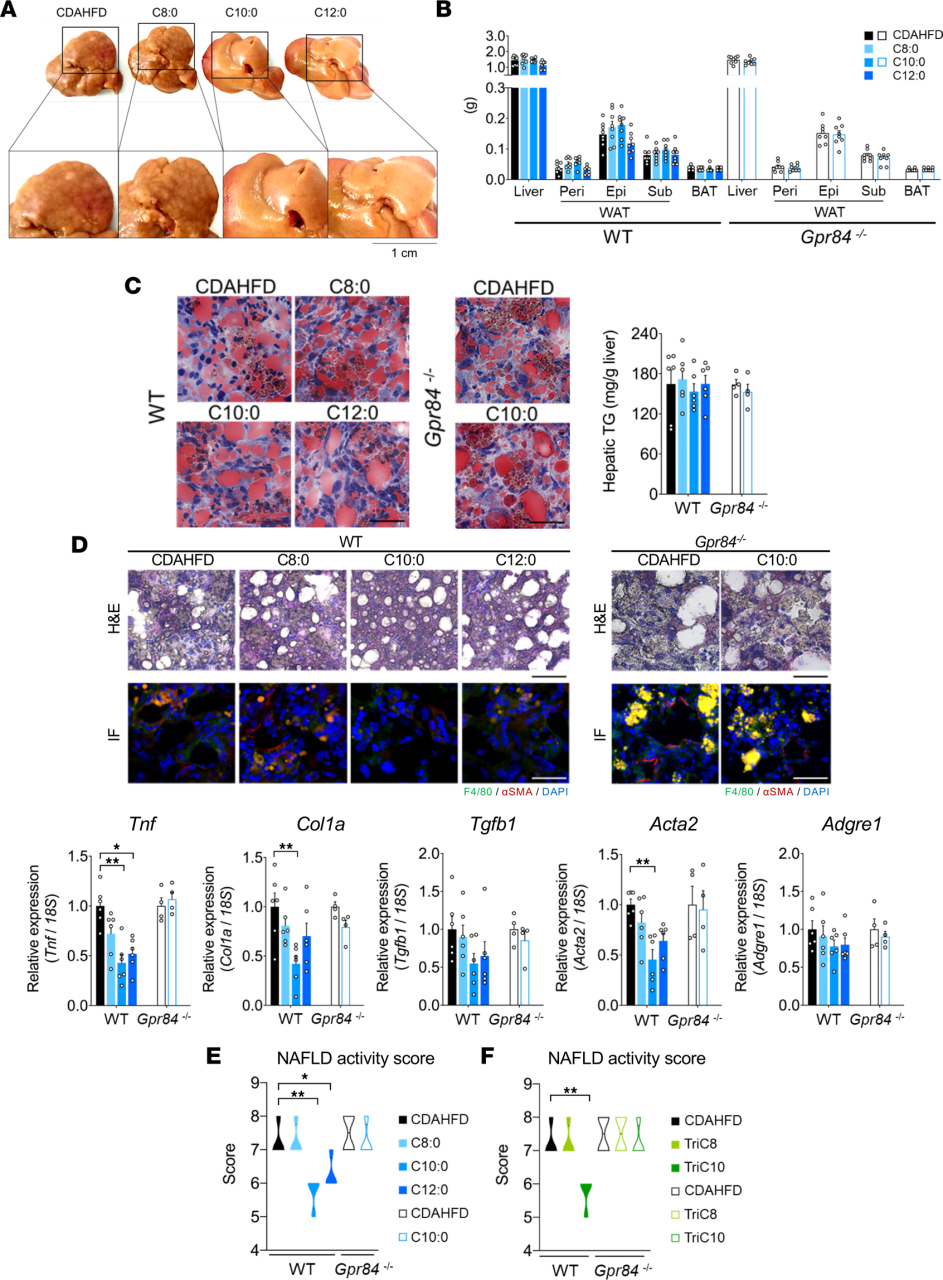
MCFA intake improves NASH progression via GPR84. (**A**) Anatomy of the liver in WT after CDAHFD feeding and MCFA-supplemented (C8:0, C10:0, and C12:0) CDAHFD feeding for 10 weeks (representative images from *n* = 8 tissues per group). (**B**) Tissue weight in WT and *Gpr84^–/–^* mice (WT, *n* = 8 tissues; *Gpr84^–/–^*, *n* = 8 tissues per group). Epi, epididymal; peri, perirenal; sub, subcutaneous; BAT, brown adipose tissue. (**C**) Oil Red O staining and hepatic TG levels (WT, *n* = 6; *Gpr84^–/–^*, *n* = 4 tissues per group). Scale bars: 50 μm. (**D**) Representative H&E staining images of the liver, and IHC of F4/80 (green), α-SMA (red), and DAPI (blue) performed in sections of the liver (upper left, WT; upper right, *Gpr84^–/–^*). Scale bars: 50 μm. Expression of inflammation- and fibrosis-related genes in the liver (lower left and right; WT, *n* = 6; *Gpr84^–/–^*, *n* = 4 samples per group). Data are represented as relative to the gene expression in CDAHFD-fed mice. (**E** and **F**) NAS (**E**) after MCFA-supplemented (C8:0, C10:0, and C12:0) CDAHFD feeding for 10 weeks and (**F**) after MCT-supplemented (TriC8 and TriC10) CDAHFD feeding for 10 weeks. *P < 0.05; **P < 0.01 (Kruskal-Wallis with Dunn’s post hoc test: **D**; ANOVA with Dunnett’s test: **E** and **F**). All data are presented as the mean ± SEM.

Finally, we investigated whether GPR84 activation could suppress NASH progression in a NASH mouse model. A choline-deficient l-amino acid–defined HFD (CDAHFD) and CCl_4_ were used to establish NASH with rapidly progressive hepatic fibrosis in mice (28). WT mice fed with the CDAHFD for 10 weeks exhibited signs of NASH and HCC (Figure 5A). Supplementation of dietary MCFAs (C8:0, C10:0, and C12:0) in CDAHFD-fed mice increased the plasma and hepatic levels of each MCFA (Supplemental Figure 10A). Interestingly, unlike in HFD-fed mice (Figure 3C), basal endogenous MCFA levels were comparable among NC-fed, CDAHFD-fed, and CCl_4_-administered mice (Supplemental Figure 10B). Although MCFA supplementation did not significantly change the liver and WAT weights, C10:0 and C12:0 supplementation in CDAHFD-fed WT mice effectively suppressed the signs of NASH and HCC (Figure 5, A and B). The hepatic TG levels were comparable between CDAHFD-fed WT and *Gpr84^–/–^* mice supplemented with dietary MCFAs (Figure 5C). The levels of the inflammatory marker *Tnf*, fibrosis markers *Col1a*, *Tgfb1*, and *Acta2*, and macrophage marker *Adgre1* were also markedly decreased by C10:0 and C12:0, but not C8:0, supplementation in the livers of CDAHFD-fed WT mice. The effects of C10:0 were abolished in *Gpr84^–/–^* mice (Figure 5D). Consequently, the NAS decreased considerably after C10:0 and C12:0, but not C8:0, supplementation in WT mice, but not in *Gpr84^–/–^* mice (Figure 5E). Thus, MCFAs, except for C8:0, markedly suppressed NASH progression via GPR84. Furthermore, among the dietary MCT oils, which are sources of MCFAs, trioctanoin (TriC8) and tridecanoin (TriC10) supplementation increased the levels of MCFA C8:0 and C10:0 in the plasma and liver, respectively (Supplemental Figure 11A). Under TriC10 supplementation, but not TriC8, the levels of inflammatory, fibrosis, and macrophage markers markedly decreased without any changes in hepatic TG levels in CDAHFD-fed WT mice, but not *Gpr84^–/–^* mice (Supplemental Figure 11, B–D). The NAS markedly dropped after TriC10 supplementation (Figure 5F). Thus, GPR84 activation by dietary MCFAs (C10:0 and C12:0, but not C8:0) markedly improves NAFLD, thereby suppressing the progression of NAFL to NASH, but not to hepatic steatosis.

### GPR84 agonists are potential NASH therapeutic drugs.

**Figure 6:**
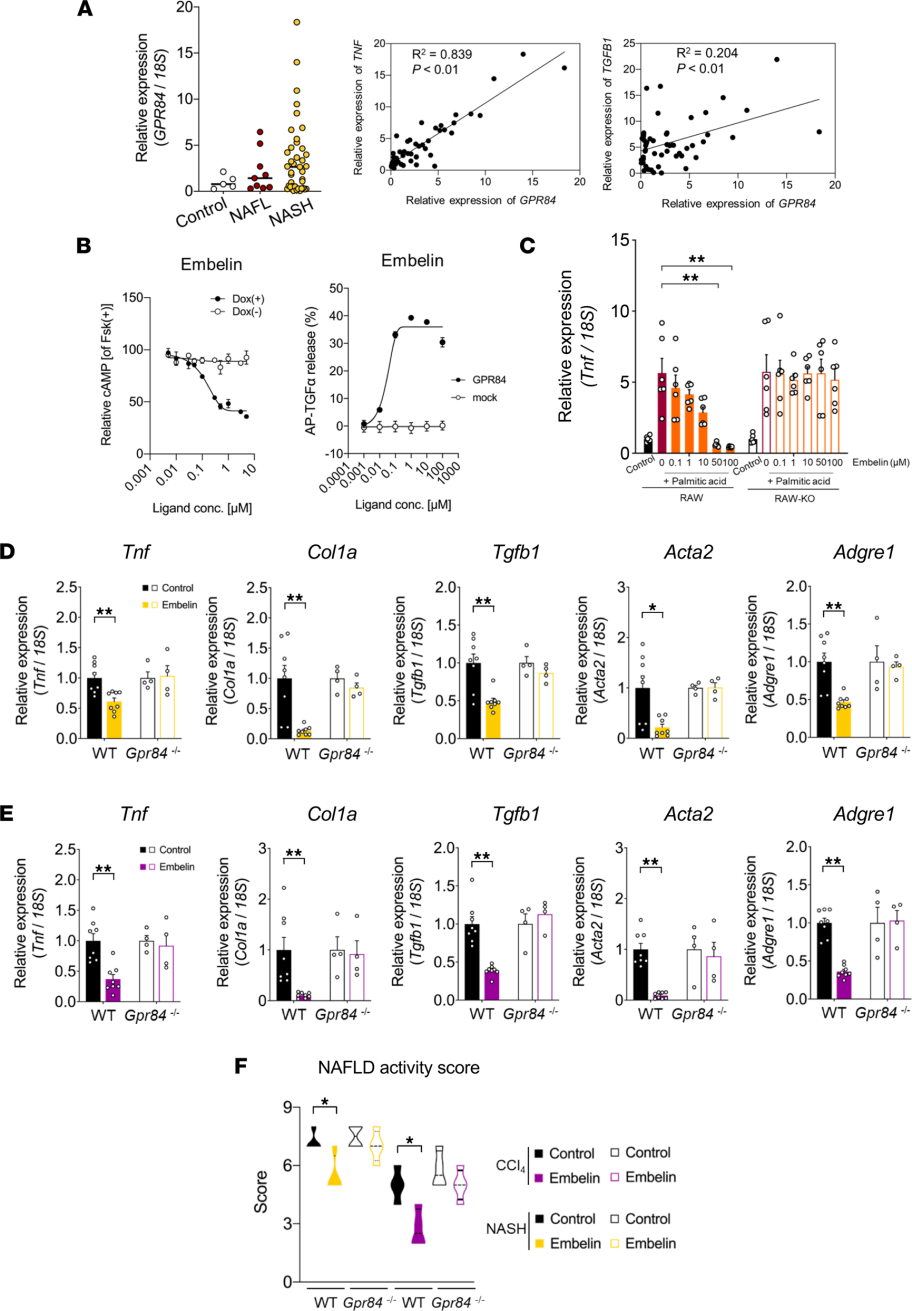
GPR84 activation improves NASH. (**A**) GPR84 expression in human liver (control, *n* = 5; NAFL, *n* = 9; NASH, *n* = 39). Pearson’s correlation coefficient between the expression levels of *GPR84* and *TNF* or *TGFB1* (*n* = 53). (**B**) Ligand affinity of embelin for GPR84 using Tet-On/Off (*n* = 6 independent cultures with Dox, from 2 biological replicates; *n* = 6 independent cultures without Dox, from 2 biological replicates) and TGF-α shedding assay (*n* = 6 independent cultures with GPR84-encoding plasmid; *n* = 6 independent cultures with mock). AP-TGFα, alkaline phosphatase-tagged TGF-α. (**C**) Antiinflammatory effect of embelin-stimulated GPR84. RAW264.7 cells and RAW-KO cells were pretreated with palmitic acid (C16:0; 200 μM) and followed by stimulation of embelin for 3 hours (*n* = 6 per group; independent experiments). Data are represented as relative to the gene expression in untreated cells. (**D**) Improvement of inflammation and fibrosis in the livers of mice fed CDAHFD for 10 weeks. Expression of inflammation- and fibrosis-related genes in the liver (WT, *n* = 8 tissues; *Gpr84*^–/–^, *n* = 4 tissues per group per group). Data are represented as relative to the gene expression in control mice (untreated with embelin). (**E**) Suppression of inflammation and fibrosis in CCl_4_-accelerated NASH. Expression of inflammation- and fibrosis-related genes in the liver (WT, *n* = 8; *Gpr84*^–/–^, *n* = 4 tissues per group). Data are represented as relative to the gene expression in control mice (untreated with embelin). (**F**) NAS after administration of the GPR84 agonist embelin. *P < 0.05; **P < 0.01 (Kruskal-Wallis with Dunn’s post hoc test: **C**; Mann-Whitney *U* test: **D**–**F** [CCl_4_]; Student’s *t* test: **F** [CDAHFD]). All data are presented as the mean ± SEM.

We confirmed that *Gpr84* expression and NASH progression increased in human livers (Figure 6A). Therefore, GPR84-selective compounds may be potential therapeutic drugs. Embelin is a known GPR84 agonist (29). In HEK293 cells expressing mouse GPR84, a tetracycline-controlled Tet-On gene expression system and TGF-α shedding assay (30) were used to confirm that embelin activated GPR84 in a dose-dependent manner (Figure 6B). Embelin, as well as C10:0, suppressed palmitate-induced increases in *Tnf* expression in a dose-dependent manner. The effects of embelin were abolished in *Gpr84^–/–^* RAW264.7 cells (Figure 6C). Hence, we administered GPR84-selective compounds in the NASH mouse model using embelin as the GPR84 agonist. Consequently, embelin markedly suppressed the levels of inflammatory, fibrosis, and macrophage markers, as well as the NAS, in both the CDAHFD-fed and CCl_4_-induced NASH mouse models (Figure 6, D–F; and Supplemental Figure 12, A and B). Thus, exogenous GPR84 stimulation markedly improved NAFLD.

## Discussion

The exact contribution of endogenous MCFAs and the receptor GPR84 in controlling metabolic syndrome was previously unclear. Herein, MCFAs showed hepatoprotective activity against dietary fat–induced NASH progression. Under HFD feeding, NASH progression was observed in HFD-fed *Gpr84^–/–^* mice. In addition to saturated fatty acid excess–mediated macrophage activation under HFD feeding, macrophage-mediated phagocytosis of fat-accumulated hepatocytes further accelerated the inflammatory response. Thus, under lipotoxic conditions, endogenous MCFAs, which are released from hepatocytes along with long-chain fatty acids, suppressed the overactivation of macrophages via GPR84, thereby protecting hepatic functions.

Metabolic disorders, such as obesity, insulin resistance, glucose intolerance, and type 2 diabetes, are significant risk factors of NASH (4, 5). We recently reported that MCFA-stimulated GPR84 activation maintains glucose homeostasis by insulin regulation via glucagon-like peptide-1 (GLP-1) secretion (25). GLP-1 also suppresses the proinflammatory and profibrotic phenotypes of macrophages, thereby suppressing NASH development (31, 32). The regulation of GLP-1 secretion via GPR84 may thus be partly related to the suppression of NASH. Thus, GPR84 functions, including differentiation of macrophages from monocytes and filtration from BM to the liver, on other organs also may influence the NASH progression. Therefore, further studies using tissue-specific GPR84-deficient mice are needed to elucidate these metabolic mechanisms.

Although it is known that MCFAs and MCTs have antiinflammatory effects and that GPR84 is coupled with inhibitory G proteins (Gi/o) (33, 34), recent in vitro studies have described GPR84 as a proinflammatory receptor (35–37). However, since these studies were conducted using only potent synthetic GPR84 agonists, the physiological activity of GPR84 remains unclear. In this study, we showed that endogenous or dietary MCFAs effectively suppress NASH progression through GPR84 as an *antiinflammatory* receptor, both in vivo and in vitro. Moreover, we confirmed the MCFA-GPR84–mediated antiinflammatory effects under lipotoxic conditions using blinded in vitro experiments. Previous in vitro studies have reported that GPR84 stimulation weakly promotes inflammation under normal or nonlipotoxic inflammatory conditions. In contrast, we showed that GPR84 stimulation suppresses inflammation under lipotoxicity-induced hyperinflammatory conditions. This contradiction may exemplify how FFARs, including GPR84, are optimal fine-tuning receptors for maintaining homeostasis by regulating biological processes and sensing nutritional states (7). Therefore, we redefine GPR84 as an immunomodulating receptor, not simply a proinflammatory receptor.

GPR84 antagonists weakly suppress NASH, and HFD feeding in *Gpr84^–/–^* mice weakly restores fibrosis, but not steatosis and inflammation (38–40). However, actual phase II clinical trials using the selective GPR84 antagonist GLPG1205 failed to demonstrate its efficacy (35, 41, 42). Furthermore, another GPR84 antagonist (PBI-4547) also acts as a GPR40/GPR120 agonist (40). Importantly, our results indicate that, although HFD feeding induced an increase in hepatic MCFA levels (as endogenous GPR84 ligands) (Figure 3C), CDAHFD feeding and CCl_4_ administration did not change hepatic MCFA levels compared with NC feeding (Supplemental Figure 10B). Furthermore, HFD-fed *Gpr84^–/–^* mice exhibited increased hepatic inflammation and fibrosis and progression to NASH compared with WT mice (Figure 2, C–E), whereas CDAHFD and CCl_4_ did not change the basal levels of inflammatory and fibrosis markers nor the NAS between WT and *Gpr84^–/–^* mice (Figures 5 and 6). Therefore, Simard et al.’s HFD-fed mouse model (40) may not alter hepatic MCFA levels, and their methods, in which 10- to 14-week-old mice were fed an HFD for 14 weeks, might not be appropriate for establishing an HFD-induced metabolic syndrome mouse model. In comparison, our method involved mice aged 7 weeks that were fed an HFD for 5 weeks, or mice aged 4 weeks that were fed an HFD for 12 weeks. Although HFD induced obesity in our mouse model, neither our CDAHFD-fed or CCl_4_-administered mice nor Simard et al.’s HFD-fed mice (40) exhibited an increase in BW. Further studies are needed to clarify the mechanism by which endogenous MCFAs are produced under HFD feeding and that of metabolic diseases and NASH progression. Nevertheless, our results indicate that exogenous GPR84 stimulation using dietary MCTs and a GPR84 agonist is effective in suppressing progression of NASH under low endogenous hepatic MCFA levels. To validate GPR84 as a therapeutic target, we suggest that GPR84 stimulation by GPR84 agonists may be a more effective strategy than developing a substitute GPR84 antagonist.

In conclusion, GPR84 deficiency under excess dietary fat intake accelerates lipotoxicity-induced macrophage overactivation, thereby promoting hepatic fibrosis to NASH. In contrast, MCFA, MCT, and GPR84 agonist administration effectively improved NASH progression by suppressing hepatic fibrosis without influencing hepatic steatosis by fat accumulation. Hence, MCFAs, either endogenously synthesized or derived from dietary MCTs, may play important roles in recognizing nutrient excess and maintaining hepatic metabolic functions through GPR84 activation. Additionally, this study formally demonstrated that orphan GPCR GPR84 is a receptor for endogenous MCFAs. Collectively, GPR84 modulation may be an effective strategy for improving the progression of NASH and HCC.

## Methods

### Animal study.

C57BL/6J, *Gpr84*^–/–^, and congenic CD45.1 mice (Sankyo Lab Service) were housed under a 12-hour light/12-hour dark cycle and fed NC (CE-2, CLEA). *Gpr84*^–/–^ mice with a C57BL/6J background were generated using the CRISPR/Cas9 system (Supplemental Figure 1, A–C). For short-term treatment, 7-week-old male mice were fed NC or an HFD with 60% kcal fat (D12492, Research Diets) for 5 weeks. For long-term treatment, 4-week-old C57BL/6J male mice were fed an HFD for 12 weeks. At least 3 groups of littermates from each dam were analyzed in individual experiments. Chronic liver injury was induced by feeding the mice with CDAHFD containing 60% kcal of fat and 0.1% of methionine (A06071302, Research Diets) (43) or MCFA- or MCT-supplemented CDAHFD (Supplemental Table 1) for 10 weeks. MCT oils were purchased from the Nisshin OilliO Group. C57BL/6J male mice that were 7–8 weeks old were treated with CCl_4_ (0.6 mL/kg body weight, diluted in corn oil and injected i.p. every 3 days) for 8 weeks to induce hepatic fibrosis (44). CDAHFD-fed or CCl_4_-treated mice were administered embelin (50 mg/kg body weight) through oral gavage once a day for 4 weeks. All efforts were made to minimize animal suffering.

### Human study.

For the analysis of *GPR84*, *TNF*, and *TGFB1* mRNA expression levels in human liver samples, a total of 53 liver samples were isolated from healthy participants, as well as patients with NAFL and NASH.

### Biochemical analyses.

Hepatic levels of TGs were analyzed using commercial kits (LabAssay Triglyceride, FUJIFILM Wako). The levels of TNF-α were measured using the Mouse TNF-alpha Quantikine ELISA Kit (R&D Systems), following the manufacturer’s instructions.

### RNA isolation and quantitative reverse transcriptase PCR.

Total RNA was extracted using an RNAiso Plus reagent (TAKARA). cDNA was transcribed using RNA as templates and Moloney murine leukemia virus reverse transcriptase (Invitrogen). Quantitative reverse transcriptase PCR analysis was performed using SYBR Premix Ex Taq II (TAKARA) and the StepOne real-time PCR system (Applied Biosystems) as described previously (10, 11). The PCR protocol was as follows: 95°C for 30 seconds, followed by 40 cycles of 95°C for 5 seconds, 58°C for 30 seconds, and 72°C for 1 minute. Each sample was tested in duplicate for the average Ct value. Relative mRNA expression was calculated after normalization to the 18S rRNA reference gene using the 2-ΔΔCt method. Primer sequences for the targeted mouse genes were as follows: *Gpr84*, 5′-AGGTGACCCGTATGTGCTTC-3′ (forward) and 5′-GTTCATGGCTGCATAGAGCA-3′ (reverse); *18S*, 5′-CTTAGAGGGACAAGTGGCG-3′ (forward) and 5′-ACGCTGAGCCAGTCAGTGTA-3′ (reverse); *Col1*α, 5′-CCTCAGGGTATTGCTGGACAAC-3′ (forward) and 5′-ACCACTTGATCCAGAAGGACCTT-3′ (reverse); *Tgfb1*, 5′-CCTGAGTGGCTGTCTTTTGACG-3′ (forward) and 5′-AGTGAGCGCTGAATCGAAAGC-3′ (reverse); *Acta2*, 5′-GTTCAGTGGTGCCTCTGTCA-3′ (forward) and 5′-ACTGGGACGACATGGAAAAG-3′ (reverse); *Tnf*, 5′-GGCAGGTCTACTTTGGAGTC-3′ (forward) and 5′-TCGAGGCTCCAGTGAATTCG-3′ (reverse); *Adgre1*, 5′-GATGTGGAGGATGGGAGATG-3′ (forward) and 5′-ACAGCAGGAAGGTGGCTATG-3′ (reverse); *Cd68*, 5′-TCCAAGATCCTCCACTGTTG-3′ (forward) and 5′-ATTTGAATTTGGGCTTGGAG-3′ (reverse); *Cd14*, 5′-GGCGCTCCGAGTTGTGACT-3′ (forward) and 5′-TACCTGCTTCAGCCCAGTGA-3′ (reverse); *Ccl2*, 5′-AATCTGAAGCTAATGCATCC-3′ (forward) and 5′-GTGTTGAATCTGGATTCACA-3′ (reverse); *Arg1*, 5′-AAAGCTGGTCTGCTGGAAAA-3′ (forward) and 5′-ACAGACCGTGGGTTCTTCAC-3′ (reverse); *Acsl1*, 5′-TGCCAGAGCTGATTGACATTC-3′ (forward) and 5′-GGCATACCAGAAGGTGGTGAG-3′ (reverse); *Acsm3*, 5′-CTTTGGCCCCAGCAGTAGATG-3′ (forward) and 5′-GGCTGTCACTGGCATATTTCAT-3′ (reverse); *Acadl*, 5′-TTTCCTCGGAGCATGACATTTT-3′ (forward) and 5′-GCCAGCTTTTTCCCAGACCT-3′ (reverse); *Acadm*, 5′-CCAGAGAGGAGATTATCCCCG-3′ (forward) and 5′-TACACCCATACGCCAACTCTT-3′ (reverse); *Acot11*, 5′-AGATCATGGCTTGGATGGAG-3′ (forward) and 5′-AAAGGCGTTATTCACGATGG-3′ (reverse); and *Acot13*, 5′-AGCAGCATGACCCAGAACCTA-3′ (forward) and 5′-GGAGCGTGCCCAGTTTATTAGTA-3′ (reverse). Primer sequences for the targeted human genes were as follows: *GPR84*, 5′-TTCAGCCCTTCTCTGTGGACA-3′ (forward) and 5′-TGCAGAAGGTGGCACCG-3′ (reverse); *TNF*, 5′-CACTAAGAATTCAAACTGGGGC-3′ (forward) and 5′- GAGGAAGGCCTAAGGTCCAC-3′ (reverse); *TGFB1*, 5′-CCCAGCATCTGCAAAGCTC-3′ (forward) and 5′-GTCAATGTACAGCTGCCGCA-3′ (reverse); and *18S*, 5′-CGCCGCTAGAGGTGAAATC-3′ (forward) and 5′-CCAGTCGGCATCGTTTATGG-3′ (reverse).

### Histological analysis.

The liver was excised and fixed overnight at 4°C in 4% paraformaldehyde. The fixed tissues were embedded in O.C.T. compound (Sakura Finetek) and sectioned into 8 μm thick sections using a cryo-microtome (Leica). H&E staining was performed using standard techniques. The lipid contents in hepatocytes were visualized using Oil Red O staining. IHC analysis was performed using antibodies against F4/80 (1:1,000; catalog ab6640, Abcam) and α-SMA (1:300; catalog 19245, Cell Signaling Technology), and the nuclei were stained with DAPI (1:5,000; catalog 10236276001, Roche), as previously described (9). Quantification of liver macrophage was quantified by counting F4/80 positive cells (green fluorescence), and total number of cells was counted based on the DAPI nuclear staining using BZ-X710 (Keyence). The sections were washed with PBS, blocked with 5% BSA in PBS, and permeabilized with 0.1% Triton X-100 (Sigma). Next, the sections were incubated with primary antibodies, followed by incubation with secondary antibodies conjugated with a fluorescent marker. Immunoreactive signals were developed using DAB staining with the Peroxidase Stain DAB Kit (Nacalai Tesque), and the sections were counterstained with Meyers hematoxylin (FUJIFILM Wako). A histopathological evaluation of NASH was performed based on the NAS and steatosis, lobular inflammation, and ballooning degeneration scores. Steatosis, lobular inflammation, and ballooning degeneration were scored on 0–3, 0–3, and 0–2 scales, respectively. Total NAS was scored as follows: 1–3, 4/5, and 6–8. NAS is shown in Supplemental Table 2.

### RNA-Seq.

RNA was extracted from the liver and BM of NC- and HFD-fed mice using an RNAiso Plus reagent (TAKARA) and RNeasy mini kit (QIAGEN). RNA-Seq libraries were generated with the TruSeq RNA Library Prep Kit (Illumina) and sequenced on an Illumina platform. Approximately 4 Gb paired-end reads of length 100 bp per sample were obtained. The RNA-Seq data were preprocessed using Trimmomatic to remove adapters or poor-quality reads (45). The quality of the trimmed sequences was then assessed using FastQC (46). The reads were aligned to the mouse reference genome (mm10) using HISAT2 (47) with the Bowtie2 aligner (48). The aligned reads were assembled using StringTie (49). The raw read counts were subjected to relative log expression normalization to obtain DEGs from all comparisons. The data were expressed as fold change using nbinomWaldTest with DESeq2. DEGs were identified based on the following 2 criteria: FDR-adjusted *P* value < 0.05 (using the Benjamini-Hochberg procedure) and |log_2_ (fold change)| > 0.5. A gene set enrichment analysis was performed using the Kyoto Encyclopedia of Genes and Genomes database (http://www.genome.jp/kegg/). The GO terms of molecular function, biological process, cellular component, and pathway were considered.

### Cell culture.

All cell lines were cultured at 37°C with 5% CO_2_. To generate Flp-In T-REx HEK293 cells (Invitrogen) expressing murine GPR84, HEK293 cells were transfected with a mixture of pcDNA5/FRT/TO-HA-mGPR84 and pOG44 using Lipofectamine reagent (Invitrogen) (Supplemental Figure 5A). The cells were cultured in DMEM supplemented with 10 μg/mL blasticidin S (Funakoshi), 100 μg/mL hygromycin B (Gibco), and 10% FBS. For the localization analysis, the cells were fixed in 4% paraformaldehyde in PBS for 10 minutes at room temperature then permeabilized with 0.2% Triton X-100 in PBS for 30 minutes at room temperature. After washing with PBS, the cells were preincubated with 1% BSA in PBS for 1 hour then probed with the primary anti-HA high-affinity antibodies (1:1,000; clone 3F10, Roche) in 1% BSA/PBS for 1 hour at room temperature. The cells were washed twice with PBS, incubated with Alexa Fluor 488–conjugated secondary antibodies (1:200; catalog A11006, Invitrogen), and observed under a fluorescence microscope. For cAMP determination, GPR84-expressing HEK293 cells were seeded in 24-well plates (1 × 10^5^ cells/well), cultured for 24 hours, and treated with or without doxycycline (10 μg/mL; Sigma) for 24 hours. The cells were treated with 2 μM forskolin (Sigma) and 500 μM of 3-isobutyl 1-methylxanthine (Sigma) to upregulate the cAMP levels. They were then stimulated with individual MCFAs (C6:0–C14:0; Nu-Chek Prep) or embelin (Cayman Chemical) for 10 minutes. The cAMP levels were determined using the cAMP ELISA kit (Cayman Chemical) following the manufacturer’s instructions.

The TGF-α shedding assay was performed as described previously (50). HEK293 cells were seeded in 6-well plates (2 × 10^5^ cells/well) and cultured for 48 hours. Plasmid transfection was performed with a mixture of 500 ng AP-TGF-α–encoding plasmid and 200 ng GPR84-encoding plasmid with or without 100 ng Gαi3-encoding plasmid. After 1 day, the transfected cells were harvested by trypsinization, pelleted by centrifugation at 190*g* for 5 minutes at room temperature, and washed once with HBSS containing 5 mM HEPES (pH 7.4). After centrifugation, the cells were resuspended in the HEPES-containing HBSS. The cell suspension was seeded in a 96-well culture plate and incubated for 30 minutes at 37°C and 5% CO_2_. The cells were treated with GPR84 ligands diluted in HBSS containing 5 mM HEPES (pH 7.4) and 0.01% (*w/v*) BSA (fatty acid–free and protease-free grade; FUJIFILM Wako) for 1 hour. AP reaction solution (10 mM *p*-nitrophenyl phosphate, 120 mM Tris-HCl [pH 9.5], 40 mM NaCl, and 10 mM MgCl_2_) was dispensed into the cell plates. Absorbance at 405 nm of the plates was measured using a microplate reader (Multiskan GO, Thermo Fisher Scientific) before and after a 1-hour incubation period at room temperature. Ligand-induced AP-TGF-α release was calculated as described previously (50).

RAW264.7 cells (mouse macrophage cell line; ATCC) were cultured in DMEM supplemented with 1% penicillin-streptomycin solution (Gibco) and 10% FBS (51). The GPR84-deficient RAW264.7 cells (RAW-KO cells) were generated using a CRISPR/Cas9-mediated homology-independent knockin system. sgRNA targeting *Gpr84* (5′-ttcgtcccaagctccgaacc-3′) was designed based on a previous report (52) and cloned into the sgRNA expression vector peSpCAS9(1.1)-2xsgRNA (Addgene plasmid 80768). RAW264.7 cells were plated in 60 mm dishes (2.5 × 10^5^ cells/dish) and cotransfected with the recombinant peSpCAS9(1.1)-2xsgRNA and pDonor-tBFPNLS-Neo (Addgene plasmid 80766) using Lipofectamine 2000 (Invitrogen). On day 2 after transfection, the cells were cultured in medium containing 250 μg/mL G418 (FUJIFILM Wako) to select the recombinant cells. At day 10 after selection, colonies grown from single cells were isolated. RAW264.7 and RAW-KO cells were stimulated with capric acid (C10:0; 0.01, 0.1, and 1 mM; Nu-Chek Prep) or embelin (0.1, 1, 10, 50, and 100 μM; Cayman Chemical) in the presence of palmitic acid (C16:0) for 12 hours. Before stimulation with these samples, the sample origin was blinded. The cells were then harvested to isolate their RNA.

AML12 cells (mouse hepatocyte cell line; ATCC) were maintained in DMEM/HAM-F12 (1:1, 3.15 g/l-glucose) (Sigma) supplemented with 1% penicillin-streptomycin solution, 10% FBS, 0.005 mg/mL insulin, 0.005 mg/mL transferrin, and 40 ng/mL dexamethasone (53). To measure the cellular MCFA contents, AML12 cells were treated with palmitic acid (C16:0) for 48 hours and harvested for liquid chromatography-mass spectrometry (LC-MS/MS) analysis. For coculture studies, long-chain fatty acid–stimulated AML12 cells were cocultured with RAW264.7 or RAW-KO cells for 72 hours and harvested for RNA isolation.

### MCFA determination.

MCFA levels in the plasma, liver, adipose tissue, muscle, cecum, and NC and HFD samples were determined following a previously described protocol with modifications (12). The samples containing an internal control (C19:0) were homogenized in methanol and mixed with chloroform and water to extract lipids. The samples were centrifuged at 2,000*g* and 17°C for 10 minutes. The supernatant containing MCFAs was collected and dried. The samples were resuspended with chloroform/methanol (1:3, v/v) and subjected to LC-MS/MS analysis using an ultra-performance LC system (UPLC, Waters) equipped with an Acquity UPLC system coupled to a Waters Xevo TQD mass spectrometer. The samples were separated on an ACQUITY UPLC BEH C18 column (2.1 × 150 mm, 1.7 μm; Waters) using a methanol gradient in 10 mM ammonium formate aqueous solution.

### Flow cytometry.

To isolate hepatic mononuclear cells and Kupffer cells, the excised livers were cut into small pieces using a razor blade and subjected to enzymatic digestion in a digestion solution (3 mM CaCl_2_, 1 mg/mL collagenase I (FUJIFILM Wako), and 1.5% BSA in HBSS) for 2 hours at 37°C. The cell suspension was passed through a 70 μm nylon mesh cell strainer (Corning). The cells were isolated using Percoll density gradient centrifugation (54). Single-cell suspensions were blocked with an Fc receptor CD16/CD32 (clone 93, BioLegend) at 4°C for 10 minutes. For flow cytometric sorting, hepatic mononuclear cells and Kupffer cells were stained with Brilliant Violet (BV) 510–conjugated anti-CD45 (clone 30-F11, BioLegend), BV711-conjugated anti-Ly6C (clone HK1.4, BioLegend), Alexa Fluor 488–conjugated anti-F4/80 (clone BM8, BioLegend), PE-conjugated anti-CX3CR1 (clone SA011F11, BioLegend), and APC-conjugated anti-CD11b (clone M1/70, BioLegend) antibodies for 30 minutes at 4°C. The cells were then washed with FACS buffer (1× PBS containing 2% FBS and 2 mM EDTA). For the transplantation studies, hepatic mononuclear cells were obtained using collagenase digestion and Percoll density gradient centrifugation. The samples were stained with PE-Cy7–conjugated anti-CD45.1 (clone A20, BD Biosciences), APC-Cy7–conjugated anti-CD45.2 (clone 104, BD Biosciences), PE-conjugated anti-Ly6C (clone HK1.4, BioLegend), FITC-conjugated anti-F4/80 (clone BM8, BioLegend), and APC-conjugated anti-CD11b (clone M1/70, BD Biosciences) antibodies. The cells were sorted using a FACSAria III cell sorter (BD Biosciences) and FACSMelody (BD Biosciences). The purity of the sorted cells was at least 95%. Flow cytometric data were analyzed using FlowJo v10 software (BD Biosciences).

### BM cell transplantation.

C57BL/6J CD45.1 mice were lethally irradiated with a dose of 10 Gy. A total of 1 × 10^7^ cells obtained from C57BL/6J or *Gpr84^–/–^* (CD45.2) mice were intravenously injected into the irradiated recipient mice. The mice were bred with water supplemented with 1 g/L neomycin and 1 g/L ampicillin for 2 weeks after transplantation. Mice with chimeric BM were fed the HFD (D12492 diet; Research Diets) for 8 weeks. The hepatocytes were isolated, and the proportion of lymphocytes and myeloid cells was calculated using flow cytometry.

### Data availability.

The source data presented in Figures 1–6, Supplemental Figures 1–12, and Supplemental Tables 1 and 2, and RNA-Seq data have been deposited into the Dryad repository (https://doi.org/10.5061/dryad.m37pvmd36).

### Statistics.

All values are presented as mean ± SEM. The violin plots depict the median, quartiles, and data range. The normality of the data was assessed by the Shapiro-Wilk test, followed by 2-tailed Student’s *t* test or the Mann-Whitney *U* test for statistical significance at 2 groups, whereas those between multiple groups (≥3 groups) were compared using 1-way ANOVA followed by Dunnett’s test or the Kruskal-Wallis test followed by the Dunn’s post hoc test. Differences were considered significant at *P* < 0.05. The FDRs of RNA-Seq data were estimated using the Benjamini-Hochberg procedure.

### Study approval.

All experimental procedures involving mice were performed according to the protocols approved by the Committee on the Ethics of Animal Experiments of the Kyoto University Animal Experimentation Committee (Lif-K21020) and the Tokyo University of Agriculture and Technology (permit 28–87). All mice were sacrificed under deep anesthesia using isoflurane. All studies were approved by the institutional review board of Kyushu University (approval 29-476, 2021-71) and performed in accordance with relevant guidelines. Written informed consent was obtained from patients at the time of recruitment, and their records were anonymized and deidentified.

## Author contributions

ROK performed the experiments, interpreted data, and wrote the paper; HN performed the experiments, interpreted data, and wrote the paper; AN performed the experiments and interpreted data; YM performed the experiments; DT performed the experiments and interpreted data; TI performed the experiments; AU performed the experiments; MT interpreted data; MK performed the experiments; MI interpreted data; HK performed the experiments and interpreted data; TT interpreted data; AI interpreted data; TS interpreted data; KH interpreted data; YO interpreted data; and JA interpreted data. IK supervised the project, interpreted data, and wrote the paper; IK also had primary responsibility for the final content. All authors read and approved the final manuscript.

## Supplementary Material

Supplemental data

## Version 1 12/08/2022

In-Press Preview

## Version 2 01/24/2023

Electronic publication
