# Supplementary material for: Medium-chain fatty acids suppress lipotoxicity-induced hepatic fibrosis via the immunomodulating receptor GPR84
Source: JCI Insight. 2023 Jan 24;8(2):e165469. doi: 10.1172/jci.insight.165469 (PMC9977302; doi:10.1172/jci.insight.165469)
Supplement: Supplemental data [file jciinsight-8-165469-s027.pdf]

Medium-chain fatty acids suppress lipotoxicity-induced hepatic fibrosis via the immunomodulating receptor GPR84

Ryuji Ohue-Kitano, Hazuki Nonaka, Akari Nishida, Yuki Masujima, Daisuke Takahashi, Takako Ikeda, Akiharu Uwamizu, Miyako Tanaka, Motoyuki Kohjima, Miki Igarashi, Hironori Katoh, Tomohiro Tanaka, Asuka Inoue, Takayoshi Suganami, Koji Hase, Yoshihiro Ogawa, Junken Aoki, and Ikuo Kimura.

Supplemental Information

Figures 1–12

Tables 1 and 2

Supplemental Figure 1

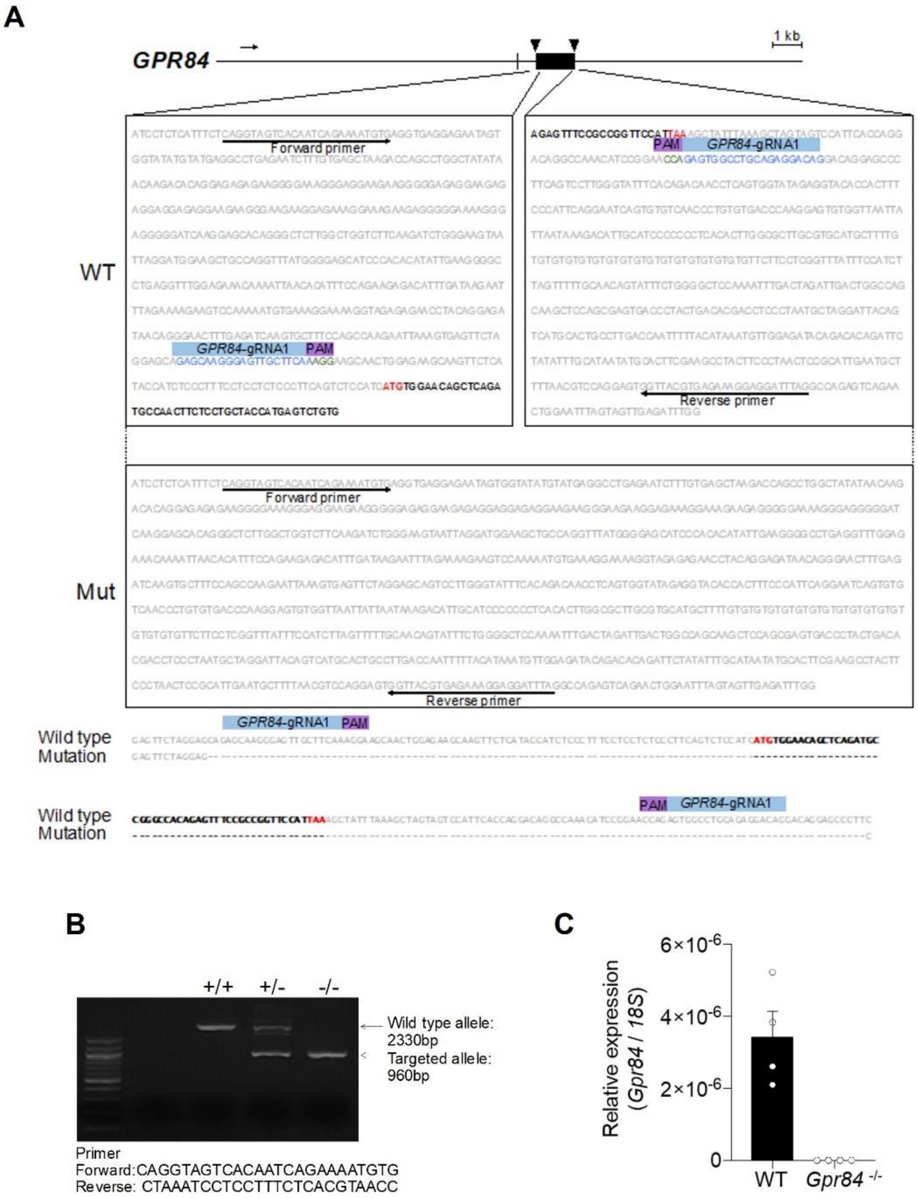

Supplemental Figure 1. Schematic representation of the structure and expression of the *Gpr84* gene. **(A)** *Gpr84* gene-knockout (*Gpr84*$^{-/-}$) mice were generated using the CRISPR/Cas9 system in wild-type C57BL/6 zygotes. Coding exon regions are shown as black boxes and words, respectively. Guide RNA (gRNA) and protospacer adjacent motif (PAM) are indicated in light blue and purple boxes, respectively. **(B)** Mouse genotypes were determined by PCR using the indicated primers to detect the wild-type and mutant alleles of *Gpr84*. **(C)** Expression of the *Gpr84* gene in bone marrow (n = 4 tissues). All data are presented as the mean ± SEM.

Supplemental Figure 2

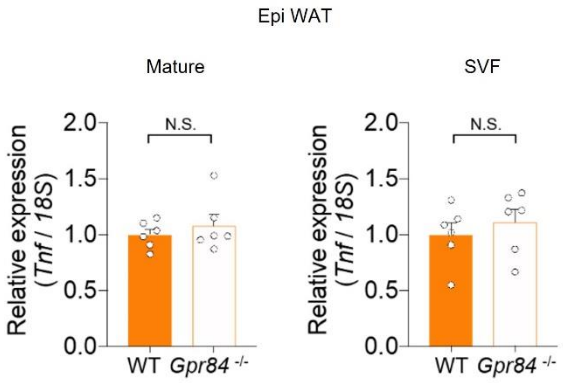

Supplemental Figure 2. ***Tnf*** **expression in** ***Gpr84***$^{-/-}$ **mice fed the HFD for 5 weeks.** Expression of *Tnf* in WAT-derived mature adipocytes and SVF (*n* = 6). Data are represented as relative to the expression of *Tnf* in WT mice. Epi WAT, epididymal white adipose tissue; SVF, stromal vascular fraction. All data are presented as the mean ± SEM. Data were statistically analyzed by Mann-Whitney U test. N.S., not significant.

Supplemental Figure 3

A

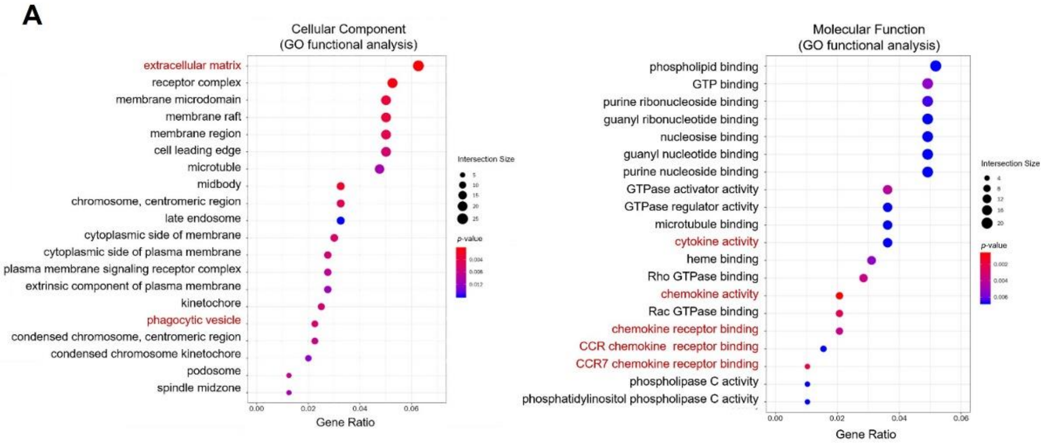

B

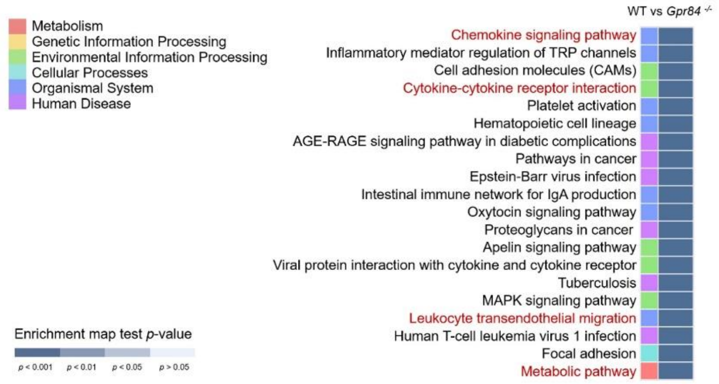

C

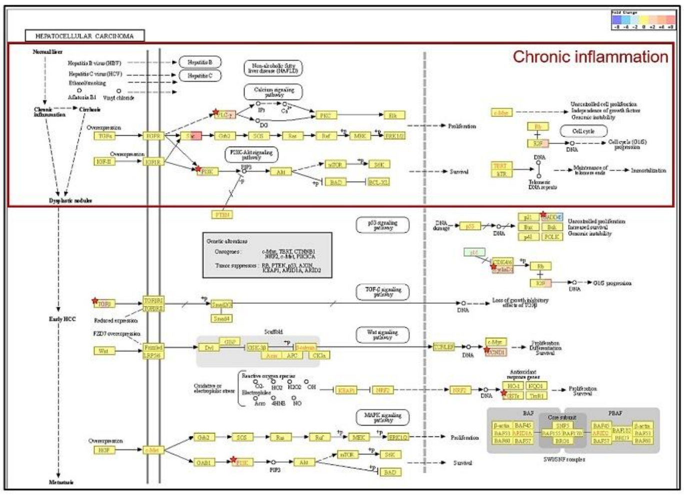

Supplemental Figure 3. Functional enrichment analysis of differentially expressed genes in the liver of WT and *Gpr84*$^{-/-}$ mice fed the HFD for 5 weeks. (A) The top 20 Gene Ontology (GO) terms related to cellular component (left) and molecular function (right). *P*-values were adjusted based on the false discovery rate (FDR). **(B)** The top 20 enriched Kyoto Encyclopedia of Genes and Genomes (KEGG) pathways of differentially expressed genes (DEGs). *P*-values were adjusted for multiple testing using Bonferroni correction. **(C)** KEGG pathway of hepatocellular carcinoma exhibits chronic inflammation (red open square). *P*-values were adjusted for multiple testing using Bonferroni correction.

Supplemental Figure 4

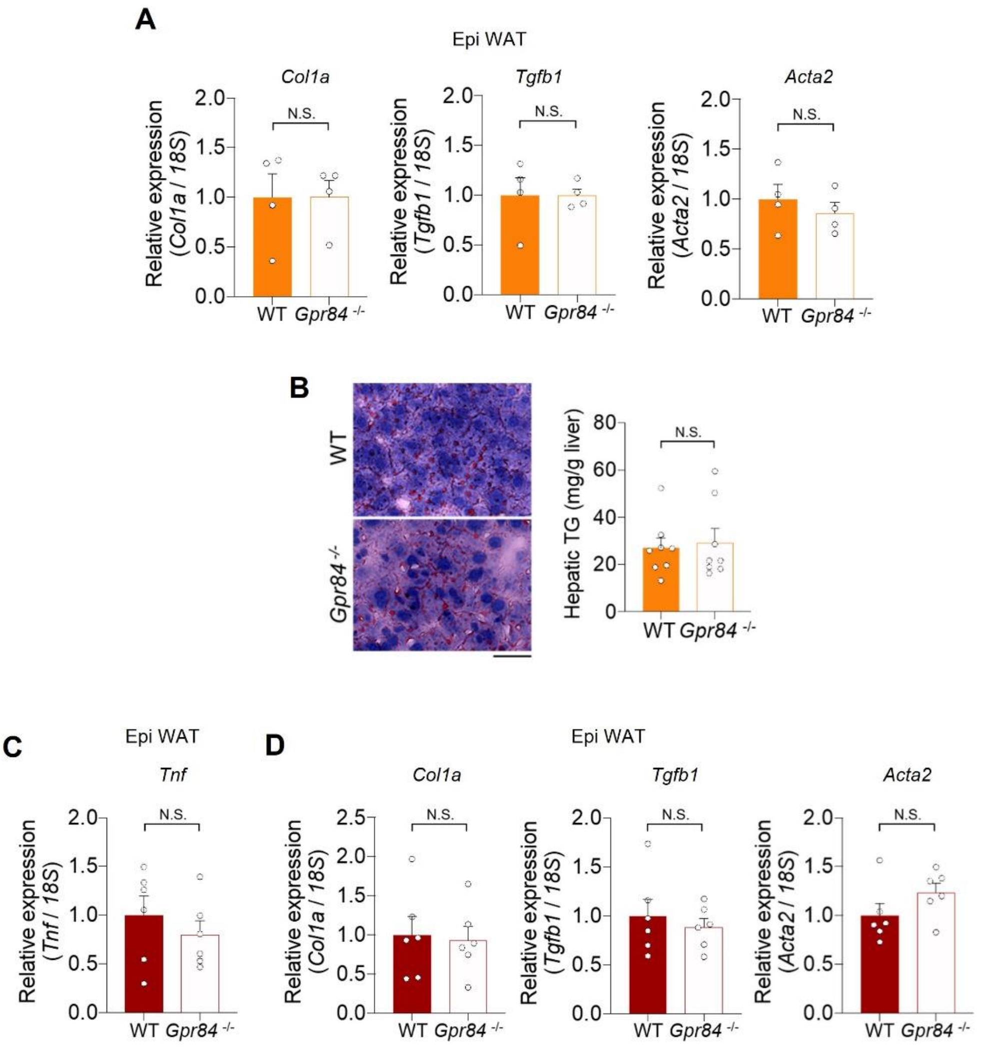

Supplemental Figure 4. GPR84 deficiency did not affect the expression of fibrosis marker genes in WAT. (A) After HFD feeding for 5 weeks, the expression of fibrosis-related genes including *Col1a* (left), *Tgfb1* (middle), and *Acta2* (right) in WAT was determined by qPCR (n = 4). Data are represented as relative to the gene expression in WT mice. **(B)** Oil Red O staining (left) and hepatic TG levels (right) (n = 8 tissues per group). Scale bars: 25 μm. **(C)** Expression of *Tnf* in WAT in WT and *Gpr84*$^{-/-}$ mice fed the HFD for 12 weeks (n = 6 samples per group). Data are represented as relative to the expression of *Tnf* in WT mice. **(D)** Expression of fibrosis marker genes in WAT: *Col1a* (left), *Tgfb1* (middle), and *Acta2* (right) (n = 6 independent experiments). Data are represented as relative to the gene expression in WT mice. All data are presented as the mean ± SEM. Data were statistically analyzed by Mann-Whitney U test. N.S., not significant.

Supplemental Figure 5

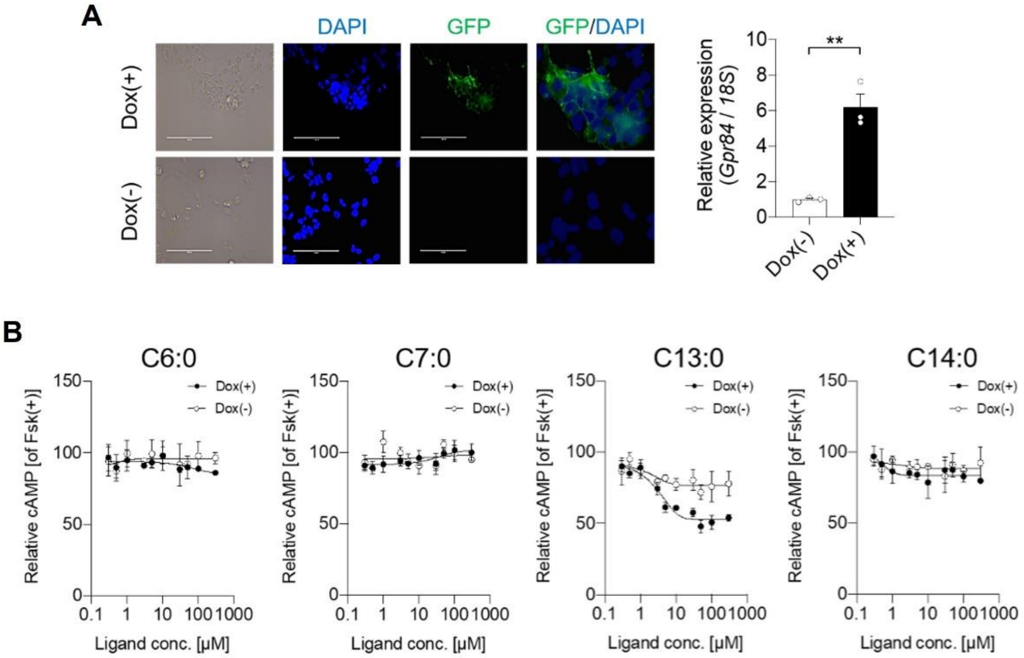

Supplemental Figure 5. Affinity of MCFAs for GPR84. (A) HEK293 cells expressing mouse GPR84 (GPR84, green; DAPI, blue) [left upper panel, Dox(+); left lower panel, Dox(-)]. Scale bars: 50 μm. Changes in *Gpr84* expression in Dox-induced HEK293 cells [right, (*n* = 3 independent experiments)]. Data are represented as relative to the expression of *Gpr84* in Dox-untreated cells. **(B)** cAMP inhibition assay for C6:0, C7:0, C13:0, and C14:0 using mouse-GPR84-expressing HEK293 cells. Cells were cultured for 24 h then treated with or without 10 μg/mL of Dox (*n* = 6 independent cultures with Dox, from two biological replicates; *n* = 6 independent cultures without Dox, from two biological replicates). All data are presented as relative to the forskolin (Fsk)-induced cAMP levels. Closed symbols represent values from cells treated with Dox, and open symbols denote untreated groups. All data are presented as the mean ± SEM. **$P < 0.01$ (Mann-Whitney U test). Dox, doxycycline.

Supplemental Figure 6

A

	Liver (nmol/g)	Muscle (nmol/g)	Adipose tissue (nmol/g)	Plasma (µM)
	ND vs HFD	ND vs HFD	ND vs HFD	ND vs HFD
**C6:0**	2.44 ± 0.27 vs 4.68 ± 1.41	1.28 ± 0.33 vs 1.34 ± 0.24	1.44 ± 0.44 vs 2.97 ± 1.00	2.61 ± 1.34 vs 2.96 ± 0.77
**C7:0**	1.87 ± 0.39 vs 1.60 ± 0.32	0.90 ± 0.15 vs 0.81 ± 0.10	1.79 ± 0.86 vs 1.15 ± 0.45	0.91 ± 0.32 vs 1.34 ± 0.42
**C8:0**	5.08 ± 0.93 vs 13.76 ± 2.25	1.33 ± 0.41 vs 1.71 ± 0.18	7.29 ± 1.06 vs 7.91 ± 0.64	3.98 ± 0.99 vs 6.22 ± 1.5
**C9:0**	1.27 ± 0.14 vs 1.28 ± 0.13	2.93 ± 0.49 vs 1.39 ± 0.27	1.91 ± 0.55 vs 2.56 ± 0.36	1.35 ± 0.37 vs 0.92 ± 0.31
**C10:0**	3.46 ± 0.53 vs 18.11 ± 2.23	1.39 ± 0.36 vs 2.16 ± 0.23	7.61 ± 0.96 vs 7.55 ± 0.62	6.57 ± 0.34 vs 14.02 ± 0.72
**C11:0**	0.17 ± 0.04 vs 0.33 ± 0.09	0.09 ± 0.04 vs 0.19 ± 0.04	0.31 ± 0.12 vs 0.18 ± 0.05	0.05 ± 0.02 vs 0.09 ± 0.03
**C12:0**	11.95 ± 1.70 vs 32.73 ± 2.56	8.73 ± 1.00 vs 8.27 ± 0.62	17.07 ± 5.10 vs 16.44 ± 2.70	9.45 ± 1.12 vs 19.74 ± 2.29
**C13:0**	0.12 ± 0.02 vs 0.15 ± 0.00	0.08 ± 0.04 vs 0.14 ± 0.04	0.18 ± 0.06 vs 0.19 ± 0.05	0.10 ± 0.03 vs 0.19 ± 0.07
**C14:0**	24.67 ± 3.29 vs 96.84 ± 6.58	16.79 ± 1.89 vs 24.71 ± 2.30	81.39 ± 20.29 vs 73.19 ± 4.86	9.34 ± 3.21 vs 12.19 ± 6.11

P value: 0.06, 0.04, 0.02, 0

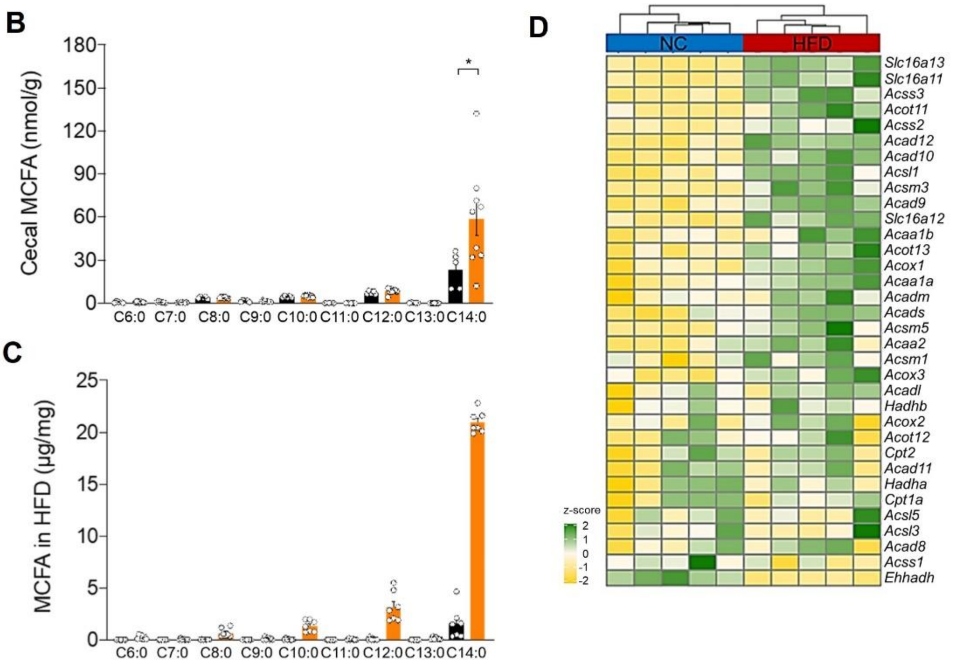

Supplemental Figure 6. Measurement of MCFAs and RNA-Seq transcriptome profiling of liver under NC and HFD feeding. (A) Measurement of MCFA contents among liver, muscle, adipose tissue, and plasma of WT mice after 5-week HFD-feeding (NC-fed group, $n = 7$; HFD-fed group, $n = 7$ tissues). **(B)** Cecal MCFA contents determined by LC/MS in WT mice fed on NC or HFD for 5 weeks ($n = 5$–9 independent experiments). **(C)** MCFA contents in NC and HFD ($n = 7$ independent experiments). **(D)** Hierarchical clustering heatmap of 34 genes related to fatty acid synthesis and β-oxidation based on DEGs. The expression levels of the DEGs are represented as FPKM-normalized log2 (fold change) values. Yellow indicates low relative expression, and green indicates high relative expression ($n = 5$ per group). All data are presented as the mean ± SEM. $^*P < 0.05$ (Mann-Whitney U test). MCFA, medium-chain fatty acid.

Supplemental Figure 7

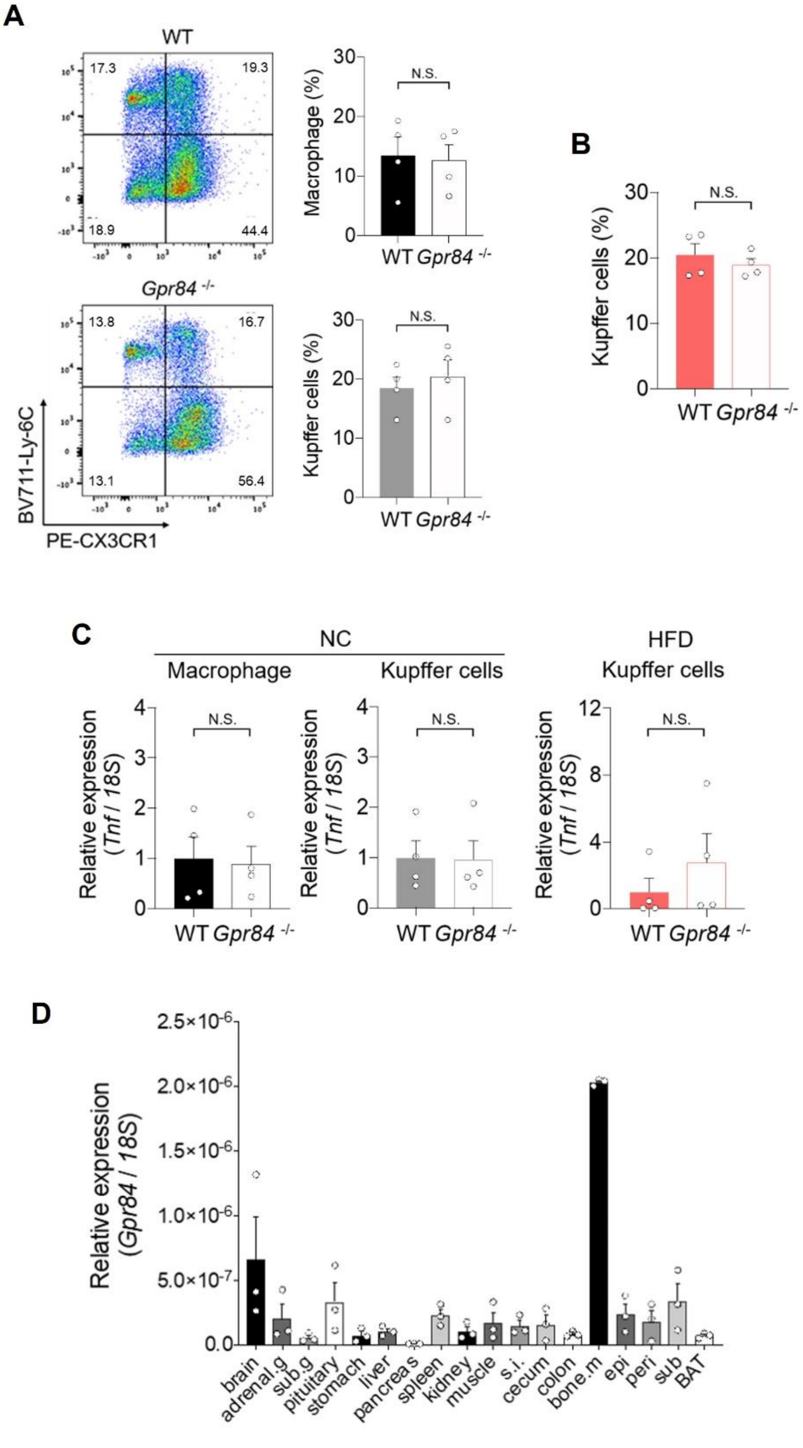

Supplemental Figure 7. Function of GPR84 in immune cells. (A) Flow cytometric analysis of the abundance of BM-derived hepatic macrophages and Kupffer cells in NC-fed WT and *Gpr84*$^{-/-}$ mice (n = 4 per group). BM-derived hepatic macrophage, $CD45^{+}Ly6C^{+}F4/80^{+}CD11b^{high}CX3CR1^{+}$; Kupffer cells, $CD45^{+}Ly6C^{-}F4/80^{+}CD11b^{low}CX3CR1^{-}$. **(B)** Flow cytometric analysis of the abundance of Kupffer cells in WT and *Gpr84*$^{-/-}$ mice fed the HFD for 12 weeks (n = 4 per group). **(C)** Comparison of *Tnf* expression between WT and *Gpr84*$^{-/-}$ mice fed NC or the HFD for 12 weeks (left, BM-derived hepatic macrophage of NC-fed mice; middle, Kupffer cells of NC-fed mice; right, Kupffer cells of HFD-fed mice; n = 4 per group). Data are represented as relative to the expression of *Tnf* in WT mice. **(D)** Expression of *Gpr84* in murine tissues (n = 3; independent experiments). adrenal.g, adrenal glands; sub.g, submandibular ganglion; s.i., small intestine; bone.m, bone marrow; epi, epididymal; peri, perirenal; sub, subcutaneous; BAT, brown adipose tissue. All data are presented as the mean ± SEM. Data were statistically analyzed by Mann-Whitney U test. N.S., not significant.

Supplemental Figure 8

A

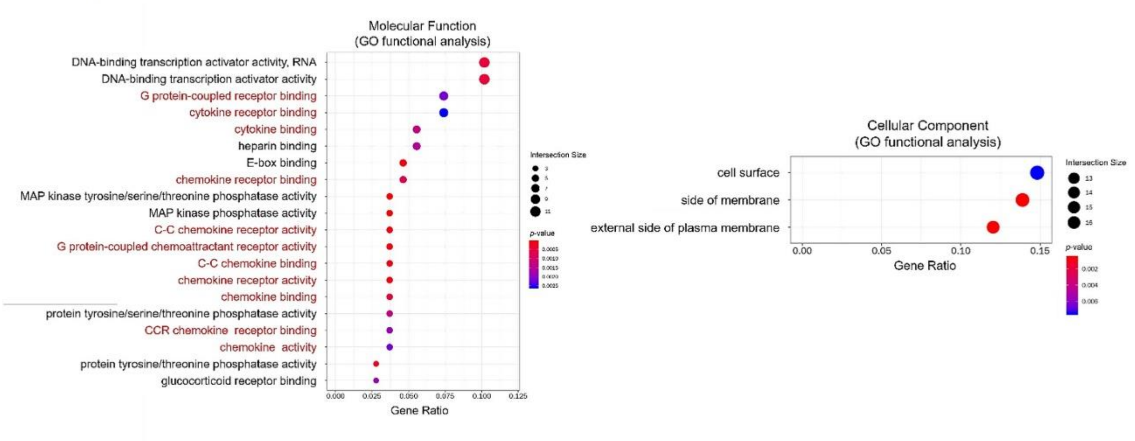

B

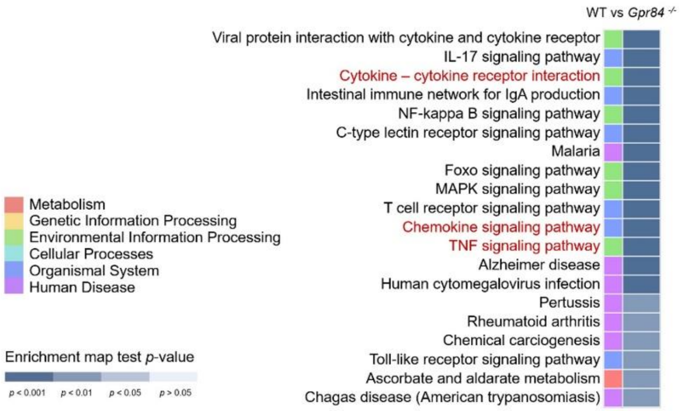

C

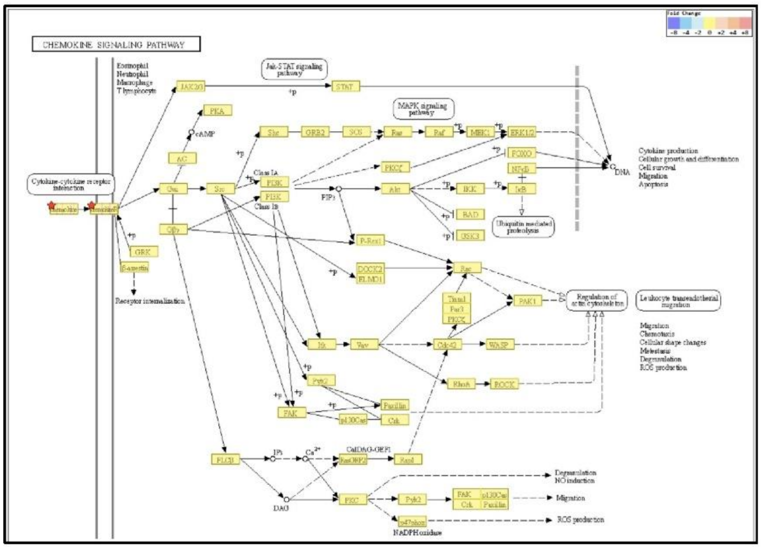

Supplemental Figure 8. Functional enrichment analysis of genes in BM of WT and *Gpr84*$^{-/-}$ mice fed the HFD for 5 weeks. (A) The top 20 GO terms related to molecular function (left) and cellular component (right). *P*-values were adjusted based on FDR. **(B)** The top 20 enriched KEGG pathways of the DEGs. *P*-values were adjusted for multiple testing using Bonferroni correction. **(C)** The KEGG pathway of the chemokine signaling pathway. *P*-values were adjusted for multiple testing using Bonferroni correction.

Supplemental Figure 9

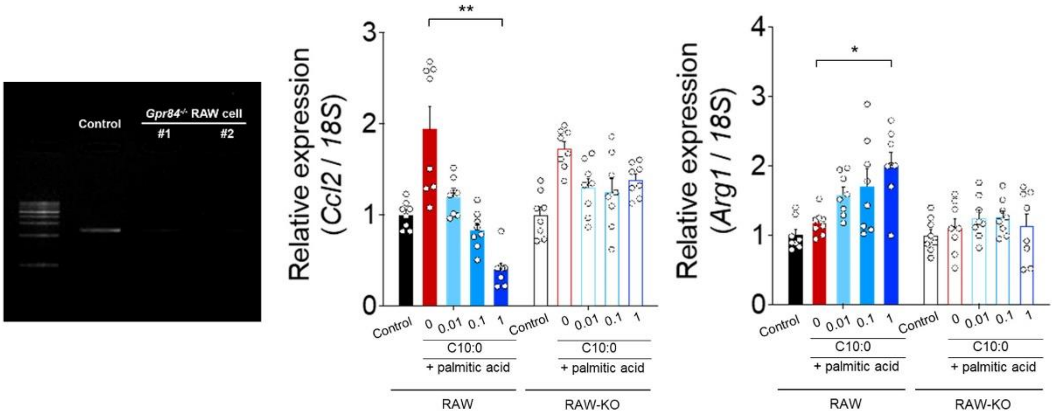

Supplemental Figure 9. Anti-inflammatory signaling by MCFA-mediated GPR84 activation in RAW264.7 cells. Anti-inflammatory effect by MCFA-mediated GPR84 stimulation. GPR84-deficient RAW264.7 cells (RAW-KO) were generated using the CRISPR/Cas9-mediated homology-independent knock-in system. *Gpr84* mRNA deficiency was screened by PCR for targeting the *Gpr84* sequence (left). RAW264.7 cells and RAW-KO cells were stimulated with C10:0 (0.01, 0.1, and 1 mM) in the presence of palmitic acid (C16:0) for 24 h. The cells were harvested to measure the expression of inflammation-related genes (right) (n = 8 per group; independent experiments). Data are represented as relative to the gene expression in untreated cells. All data are presented as the mean ± SEM. $^{**}P < 0.01$; $^{*}P < 0.05$ (Kruskal Wallis with Dunn's post-hoc test).

Supplemental Figure 10

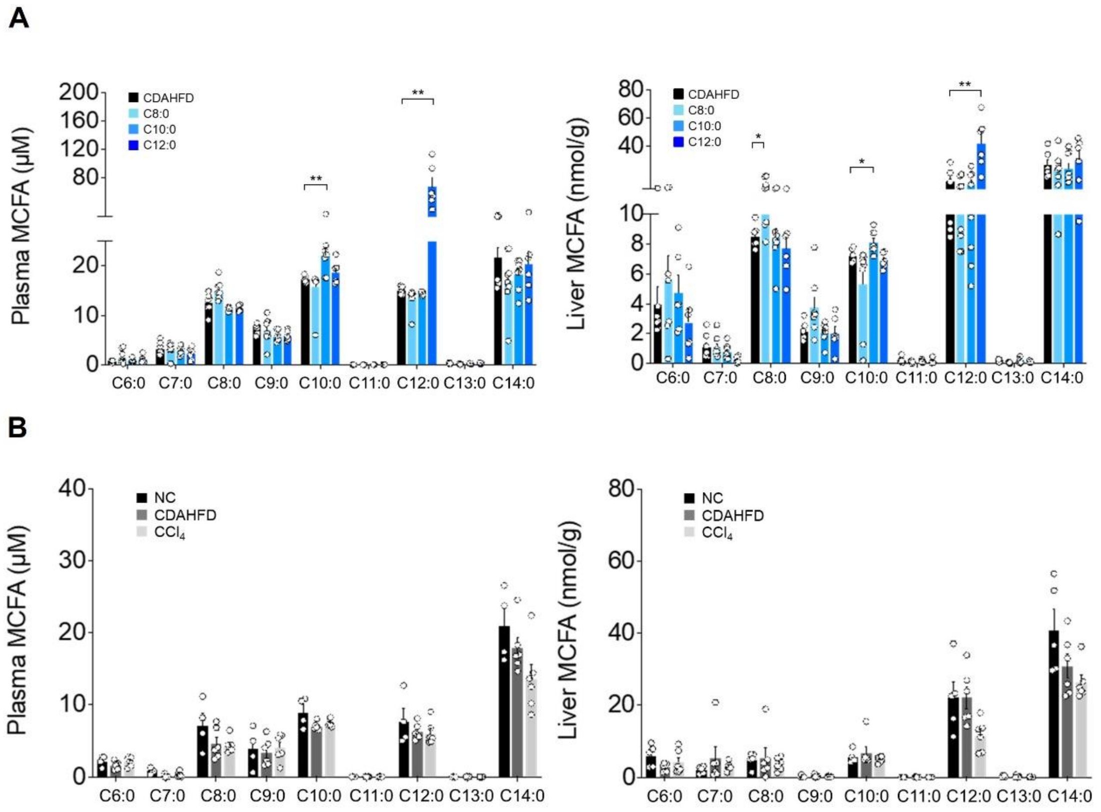

Supplemental Figure 10. Plasma and liver MCFA levels by MCFA supplementation. (A) MCFA concentration in the plasma (left), and liver (right) of mice fed the CDAHFD and MCFA (C8:0, C10:0, and C12:0)-supplemented CDAHFD (*n* = 6–8 animals per group). **(B)** MCFA concentration in the plasma (left), and liver (right) of mice fed NC or the CDAHFD, or administered CCl_4 (*n* = 4–6 animals per group). All data are presented as the mean ± SEM. $^{**}P < 0.01$; $^{*}P < 0.05$ (Mann-Whitney U test). NC, normal chow; CDAHFD, choline-deficient L-amino acid-defined high-fat diet.

Supplemental Figure 11

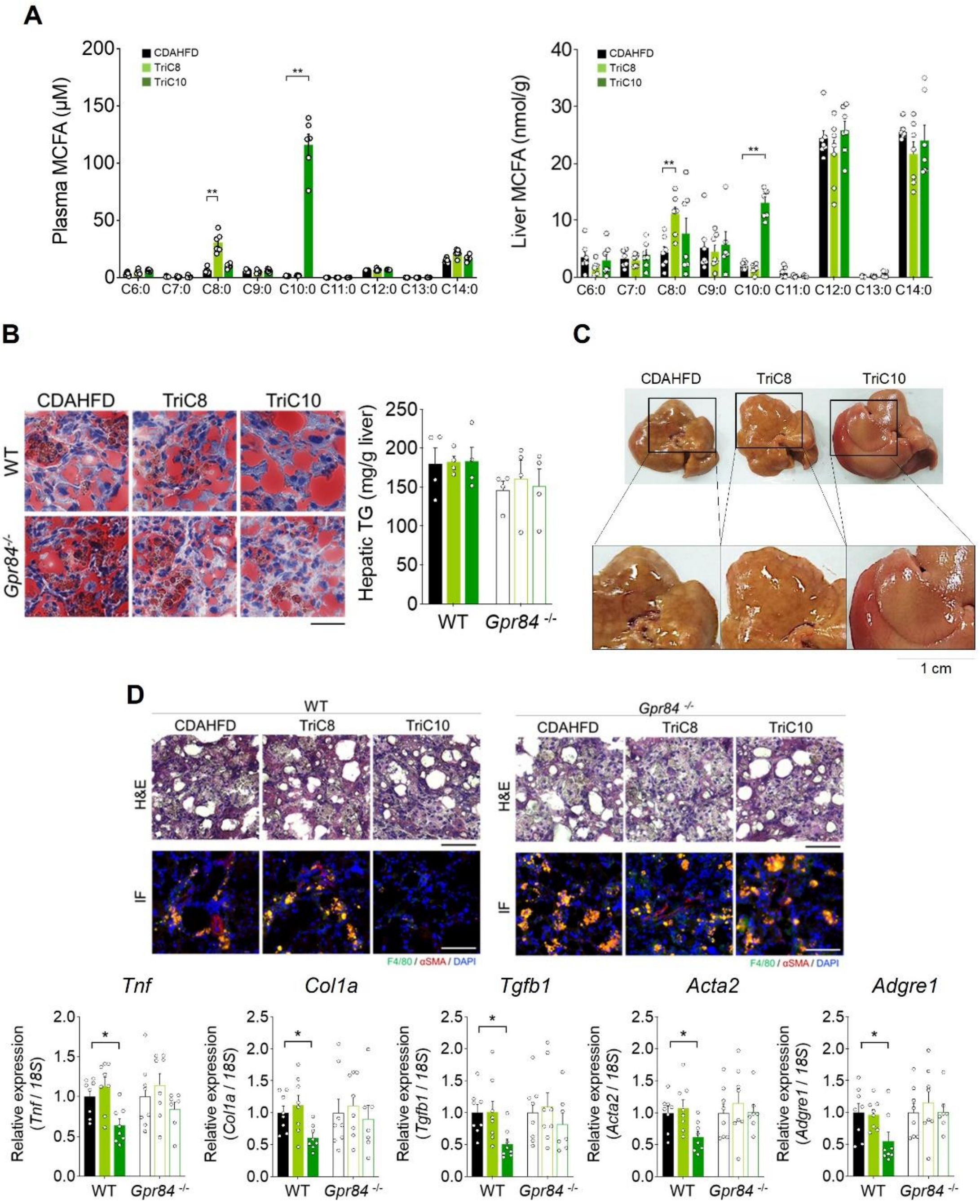

Supplemental Figure 11. MCT intake effectively ameliorates CDAHFD-induced inflammation and fibrosis via GPR84. (A) MCFA concentration in the plasma (left) and liver (right) of mice fed the CDAHFD and MCT (TriC8 and TriC10)-supplemented CDAHFD (*n* = 6–7 samples per group). **(B)** Hepatic TGs levels (right) and Oil Red O staining (left) (*n* = 4 tissues per group). Scale bars: 50 µm. **(C)** Representative anatomical images of the liver. **(D)** Representative H&E staining images of the liver, and immunohistochemistry of F4/80 (green), αSMA (red), and DAPI (blue), performed in sections of the liver tissue (upper left, WT; upper right, $Gpr84^{-/-}$). Scale bars: 50 µm. Expression of inflammation- and fibrosis-related genes in the liver (lower, *n* = 7–8 tissues per group). Data are represented as relative to the gene expression in mice fed the CDAHFD. All data are presented as the mean ± SEM. $^{**}P < 0.01$; $^{*}P < 0.05$ (Mann-Whitney U test: A; Kruskal Wallis with Dunn‘s post-hoc test: D). αSMA, α-smooth muscle actin.

Supplemental Figure 12

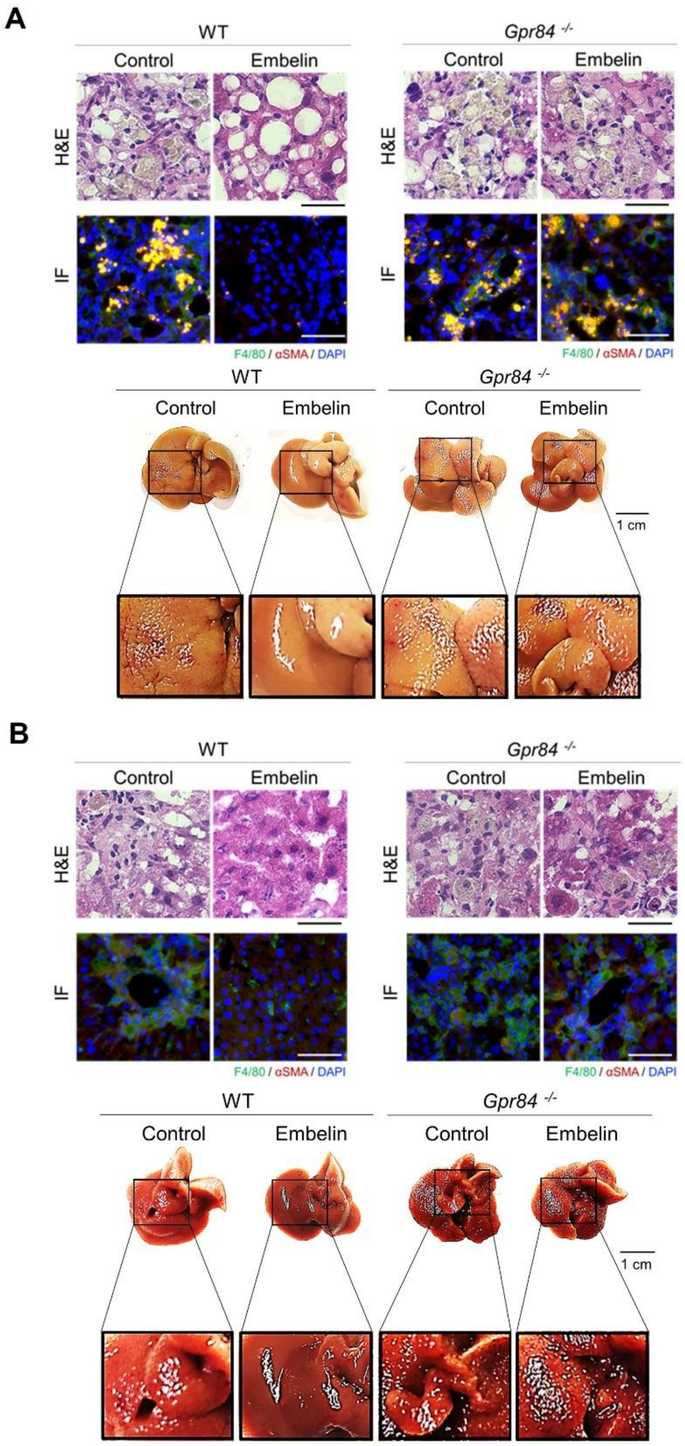

Supplemental Figure 12. GPR84 agonist suppresses NASH progression. (A) Improvement of inflammation and fibrosis in the liver of mice fed the CDAHFD for 10 weeks. Representative H&E staining images of the liver, and immunohistochemistry of F4/80 (green), αSMA (red), and DAPI (blue), performed in sections of the liver (upper left, WT; upper right, *Gpr84*$^{-/-}$). Scale bars: 50 μm. Representative anatomical images of the liver (lower left, WT; lower right, *Gpr84*$^{-/-}$). **(B)** Suppression of inflammation and fibrosis in carbon tetrachloride (CCl_4)-accelerated NASH. Representative H&E staining images of the liver, and immunohistochemistry of F4/80 (green), αSMA (red), and DAPI (blue), performed in sections of the liver (upper left, WT; upper right, *Gpr84*$^{-/-}$). Scale bars: 50 μm. Representative anatomical images of the liver (lower left, WT; lower right, *Gpr84*$^{-/-}$). αSMA, α-smooth muscle actin.

Supplemental Table 1.

Compositions of choline-deficient L-amino acid-defined high-fat diet (CDAHFD).

	Control	MCFA			MCT	
**Ingredient**	**CDAHFD**	**C8:0**	**C10:0**	**C12:0**	**TriC8**	**TriC10**
L-Isoleucine	1.010	0.960	0.960	0.960	1.010	1.010
L-Leucine	2.090	1.986	1.986	1.986	2.090	2.090
L-Lysine	1.750	1.663	1.663	1.663	1.750	1.750
L-Methionine	0.110	0.105	0.105	0.105	0.110	0.110
L-Cystine	0.560	0.532	0.532	0.532	0.560	0.560
L-Phenylalanine	1.110	1.055	1.055	1.055	1.110	1.110
L-Tyrosine	1.220	1.159	1.159	1.159	1.220	1.220
L-Threonine	0.950	0.903	0.903	0.903	0.950	0.950
L-Tryptophan	0.280	0.266	0.266	0.266	0.280	0.280
L-Valine	1.230	1.169	1.169	1.169	1.230	1.230
L-Histidine	0.610	0.580	0.580	0.580	0.610	0.610
L-Arginine	0.790	0.751	0.751	0.751	0.790	0.790
L-Alanine	0.670	0.637	0.637	0.637	0.670	0.670
L-Aspartic Acid	1.600	1.520	1.520	1.520	1.600	1.600
L-Glutamic Acid	5.050	4.798	4.798	4.798	5.050	5.050
Glycine	0.400	0.380	0.380	0.380	0.400	0.400
L-Proline	2.350	2.233	2.233	2.233	2.350	2.350
L-Serine	1.320	1.254	1.254	1.254	1.320	1.320
Maltodextrin 10	17.21	16.35	16.35	16.35	17.21	17.21
Sucrose	9.10	8.65	8.65	8.65	9.10	9.10
Cellulose, BW200	6.61	6.28	6.28	6.28	6.61	6.61
Soybean Oil	3.31	3.14	3.14	3.14	3.31	3.31
Lard	32.41	30.79	30.79	30.79	14.41	14.41
C8:0	-	5.00	-	-	-	-
C10:0	-	-	5.00	-	-	-
C12:0	-	-	-	5.00	-	-
TriC8	-	-	-	-	18.00	-
TriC10	-	-	-	-	-	18.00
Mineral Mix S10026	1.32	1.25	1.25	1.25	1.32	1.32
DiCalcium Phosphate	1.72	1.63	1.63	1.63	1.72	1.72
Calcium Carbonate	0.73	0.69	0.69	0.69	0.73	0.73
Potassium Citrate	2.18	2.07	2.07	2.07	2.18	2.18
Sodium BiCarbonate	0.99	0.94	0.94	0.94	0.99	0.99
Vitamin Mix V10001	1.32	1.25	1.25	1.25	1.32	1.32
Total [gm(%)]	100.0	100.0	100.0	100.0	100.0	100.0

Supplemental Table 2.

NAFLD activity score (NAS). (A) HFD-fed *Gpr84*$^{-/-}$ mice (n = 4 per group). **(B)** HFD-fed bone marrow chimeric mice (n = 4 per group). **(C)** MCFA-supplemented CDAHFD-fed mice (n = 4 per group). **(D)** MCT-supplemented CDAHFD-fed mice (n = 4 per group). **(E)** CDAHFD-fed or CCl_4-treated mice administered with embelin (n = 4 per group).

(A) NAFLD activity score

	Steatosis	Lobular inflammation	Hepatocyte ballooning
**WT**	2	1.5 ± 0.25	0.25 ± 0.22
***Gpr84*$^{-/-}$**	2	2.5 ± 0.25	0.75 ± 0.22

(B) NAFLD activity score

	Steatosis	Lobular inflammation	Hepatocyte ballooning
**WT**	2	1.5 ± 0.25	0.25 ± 0.22
***Gpr84*$^{-/-}$**	2	2.0 ± 0.35	1.25 ± 0.41

(C) NAFLD activity score

		Steatosis	Lobular inflammation	Hepatocyte ballooning
**WT**	**CDAHFD**	3	3	1.25 ± 0.22
	**C8:0**	3	3	1.25 ± 0.22
	**C10:0**	3	2.5 ± 0.25	0.25 ± 0.22
	**C12:0**	3	2.75 ± 0.22	0.5 ± 0.25
***Gpr84*$^{-/-}$**	**CDAHFD**	3	3	1.5 ± 0.25
	**C10:0**	3	3	1.25 ± 0.22

(D) NAFLD activity score

		Steatosis	Lobular inflammation	Hepatocyte ballooning
**WT**	**CDAHFD**	3	3	1.25 ± 0.22
	**TriC8**	3	3	1.25 ± 0.22
	**TriC10**	3	2.5 ± 0.25	0.25 ± 0.22
***Gpr84*$^{-/-}$**	**CDAHFD**	3	3	1.5 ± 0.25
	**TriC8**	3	3	1.5 ± 0.25
	**TriC10**	3	3	1.25 ± 0.22

(E) NAFLD activity score

			Steatosis	Lobular inflammation	Hepatocyte ballooning
CDAHFD	WT	Control	3	3	1.25 ± 0.22
		Embelin	3	1.5 ± 0.25	1.0 ± 0.35
	Gpr84$^{-/-}$	Control	3	3	1.5 ± 0.25
		Embelin	3	3	1.0 ± 0.35
CCl_4	WT	Control	1.25 ± 0.22	2.5 ± 0.25	1.25 ± 0.22
		Embelin	0.75 ± 0.41	2	0
	Gpr84$^{-/-}$	Control	2	2.5 ± 0.25	1.25 ± 0.22
		Embelin	1.5 ± 0.25	2.5 ± 0.25	1.0 ± 0.35
